# Defining thresholds of sustainable impact on benthic communities in relation to fishing disturbance

**DOI:** 10.1038/s41598-017-04715-4

**Published:** 2017-07-14

**Authors:** G. I. Lambert, L. G. Murray, J. G. Hiddink, H. Hinz, H. Lincoln, N. Hold, G. Cambiè, M. J. Kaiser

**Affiliations:** 1 School of Ocean Sciences, Bangor University, Askew Street, Menai Bridge, LL59 5AB UK; 2 IMEDEA, Mediterranean Institute for Advanced Studies (UIB-CSIC), C/Miquel Marquès, 21, 07190 Esporles Illes Balears, Spain

## Abstract

While the direct physical impact on seabed biota is well understood, no studies have defined thresholds to inform an ecosystem-based approach to managing fishing impacts. We addressed this knowledge gap using a large-scale experiment that created a controlled gradient of fishing intensity and assessed the immediate impacts and short-term recovery. We observed a mosaic of taxon-specific responses at various thresholds. The lowest threshold of significant lasting impact occurred between 1 and 3 times fished and elicited a decrease in abundance of 39 to 70% for some sessile epifaunal organisms (cnidarians, bryozoans). This contrasted with significant increases in abundance and/or biomass of scavenging species (epifaunal echinoderms, infaunal crustaceans) by two to four-fold in areas fished twice and more. In spite of these significant specific responses, the benthic community structure, biomass and abundance at the population level appeared resilient to fishing. Overall, natural temporal variation in community metrics exceeded the effects of fishing in this highly dynamic study site, suggesting that an acute level of disturbance (fished over six times) would match the level of natural variation. We discuss the implications of our findings for natural resources management with respect to context-specific human disturbance and provide guidance for best fishing practices.

## Introduction

An Ecosystem-Based Approach to Fisheries (EAF)^1^ takes account of the interaction between exploited species and their ecosystems. EAF is embodied in legislation such as the U.S. Magnuson-Stevens Fisheries Conservation and Management Act in relation to the effects of fishing on Essential Fish Habitat and in Europe through the Marine Strategy Framework Directive (MSFD)^2^, which seeks to define targets for ‘Good Environmental Status’ (GES)^3^. Fishing impacts on seabed ecosystems can be reduced by spatial and temporal closures^4^. However, such approaches often do not account for displacement effects and can potentially lead to worse outcomes if fishing is displaced to areas where the ecosystem components are more sensitive to disturbance^5^. The quantification of fishing intensity thresholds for ecosystem components other than the target species (below which the maintenance or recovery of ecosystems is not impeded by fishing activity) would offer the possibility of a more sophisticated management. The latter approach could be implemented with real-time incentives such as habitat quotas that promote fishing behaviours that minimise their associated impacts^6^.

Studies that have quantified the impact of fishing on benthic communities have used small-scale “Before-After Control-Impact” (BACI) experiments^7^ or have been large-scale long term studies of chronic fishing activities by commercial vessels. In general, smaller-scale studies give relatively short recovery times while larger-scale studies estimate recovery times from <3 years^8, 9^ to 5–10 years^10, 11^. The spatial scale of disturbance and proximity of potential recruits appear to be important factors that explain the mechanism of recovery^12^. Recovery rate is habitat- and fishing gear- dependent^13^. Recovery times following scallop dredging on sand and gravel are longer than for beam trawling or otter trawling^13^, with scallop dredging having the greatest negative impact across all habitat types^11^. Typically, fishing is likely to have a prolonged negative effect on communities and habitats in areas that experience low natural disturbance but reduced effects in more dynamic habitats^14, 15^. Some habitats are highly sensitive to fishing disturbance with a very limited capacity for recovery, e.g. biogenic habitats such as maerl beds, and reef-forming biota such as bivalves, sponges and corals^16, 17^. In contrast, areas subjected to high natural disturbance, such as shallow wave-swept areas, are characterized by communities adapted to these more disturbed conditions and tend to be species-poor and dominated by small short-lived species, and are therefore expected to be more resilient to anthropogenic disturbance^18–20^. van Denderen *et al*.^15^ showed that the functional composition of benthic species in fished and naturally disturbed areas are similar, and that fishing had no effect on functional composition in naturally disturbed areas. While it is clear that bottom-fishing disturbance can alter marine habitats and ecosystems, if we understand the pressure/state relationship it should be possible to implement management measures that minimize the impacts of fishing without the need to preclude fishing altogether.

**Figure 1:**
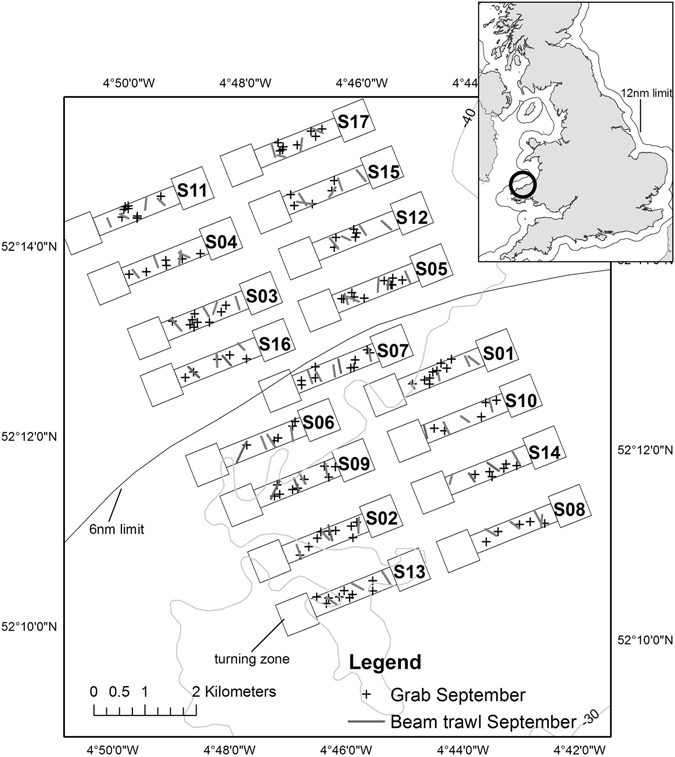
Map of the experimental area located within Cardigan Bay SAC, Wales, UK. Each site consisted of two turning zones and a fishing corridor in between. Sites are numbered in increasing order of designated fishing intensity (See Table 1). Sampling locations (+) for grabs and lines for trawls are shown for the September 2014 survey. The 6 nautical mile limit separates two distinct management zones in Wales. Within 6 nm, only vessels towing less than four dredges a side were allowed to operate, whereas the offshore sites could be fished with up to 7 dredges a side (created with ArcGIS 10.5, http://www.esri.com/).

The aim of the present study was to quantify the effect of, and recovery from, different intensities of fishing (scallop dredging) on the benthic communities and habitat characteristics. We performed a BACI experiment on an unprecedented scale that aimed to mimic the patterns of commercial scallop dredging at the spatial scale that occurs in the fishery using realistic fishing intensity levels (executed by commercial fishing vessels) (Fig. 1, Table 1). The experiment was performed within a marine protected area and therefore avoided the confounding effects of previous fishing.

**Table 1:** Summary of experimental design and data collection over all 3 surveys.

Site	FI aimed for (number of times fished)		FI achieved (number of times fished)	Number of grab samples		Number of BT samples	Depth range (m)	Texture (in fraction of number of samples collected per site)	
	*Passes*	*FI*		*Collected*	*Processed*			*Sand*	*Gravel*
S01	0	0	0.0	27	—	12	32.4–43.3	0.19	0.81
S02	0	0	0.0	26	24	14	31.8–43.4	0.12	0.88
S03	0	0	0.0	27	21	12	35.9–45.2	0.66	0.33
S04	123	0.2	0.3	16	13	14	39–45.3	0.47	0.53
S05	172	0.35	0.2	26	—	13	38.6–43.7	0.32	0.68
S06	245	0.5	0.5	15	—	11	34.3–42.6	0.47	0.53
S07	348	0.7	0.0	22	19	13	38.2–43.2	0.64	0.36
S08	491	1	1.1	15	14	11	28.7–37.3	0.72	0.28
S09	619	1.3	1.2	26	21	12	33–40.9	0.56	0.44
S10	781	1.6	1.6	16	—	13	35.6–39.6	0.25	0.75
S11	982	2	1.9	24	14	12	41–45.9	0.92	0.08
S12	1237	2.5	2.3	15	15	10	36.9–40	0.93	0.07
S13	1556	3.2	3.1	27	21	10	33.4–45.1	0.39	0.61
S14	1964	4	3.8	16	16	12	31.9–42.6	0.21	0.79
S15	2474	5	3.9	16	15	12	37.9–40.6	0.69	0.31
S16	3117	6.3	5.3	16	15	13	36.8–43.7	0.50	0.50
S17	3928	8	6.1	26	24	12	36.1–45.9	0.68	0.32

FI is the Fishing Intensity. Passes = the number of single dredge tows expected to achieve a specific FI. Number of grabs processed corresponds to grabs that have been analyzed for fauna. BT indicates the number of beam trawl samples. See map Fig. 1. Note that the design was unbalanced and not all grab samples were processed due to logistical constraints.

The Cardigan Bay Special Area of Conservation (SAC) (960 km^2^), Wales, UK, was established in 2004. The SAC is designed to protect bottlenose dolphin, grey seal, sea lamprey, subtidal sandbanks and cobble reefs^21^. Previously, scallop dredging activity in this area was not managed other than between the 0–3 nautical mile boundary within which all scallop dredging was prohibited. However, since 2009, the scallop fishery that occurred in the SAC has been restricted and 15% of the area is now open to scallop dredgers during 6 months of the year (see Sciberras *et al*.^21^ for a full description of the management history of the site). The present study was conducted in the part of the SAC from which scallop dredging has been excluded since 2009.

We conducted a BACI fishing intensity gradient experiment in 2014, which comprised three scientific surveys, one directly prior to scallop dredging (hereafter termed ‘fishing’), one directly post-fishing and a final survey four months post-fishing. A total of ca. 1,100 h of fishing was undertaken by commercial scallop dredgers (or 12,000 dredge hours) during which 30 tonnes of scallop meat were yielded. The experiment aimed to quantify the impact of different intensities of fishing on infauna and epifauna and to identify fishing intensity thresholds beyond which the state or recovery of the benthos was affected significantly.

## Results

### Benthic Ecosystem Descriptors

The benthic community was characterized by a low number of individuals spread across a similarly low number of species. A total of 161 families of infaunal taxa and 172 epifaunal species (some at the genus level) were identified over the three surveys (listed in Tables S1.1 and S1.2). Before fishing, there was an average of 35.4 epifaunal species per haul (ca. 550 m^−2^) [34.2 in sand, 35.9 in gravel] with an average of 38.8 individuals per 100 m^2^ weighing 468 g 100 m^−2^ [40.2 ind. and 490 g/100 m^2^ in sand, 36.6 ind. and 413 g/100 m^2^ in gravel] (excluding *Pecten maximus* and *Ophiothrix fragilis*). In the infaunal samples, there was an average of 11.1 family-level taxa [10.4 in sand, 11.4 in gravel] and 35.9 individuals weighing 8.9 g 0.1 m^−2^ [28.5 ind. and 10.1 g/0.1 m^−2^ in sand, 44.9 ind. and 7.1 g/0.1 m^−2^ in gravel].

### Impact of fishing gradient on overall abundance and biomass

**Figure 2:**
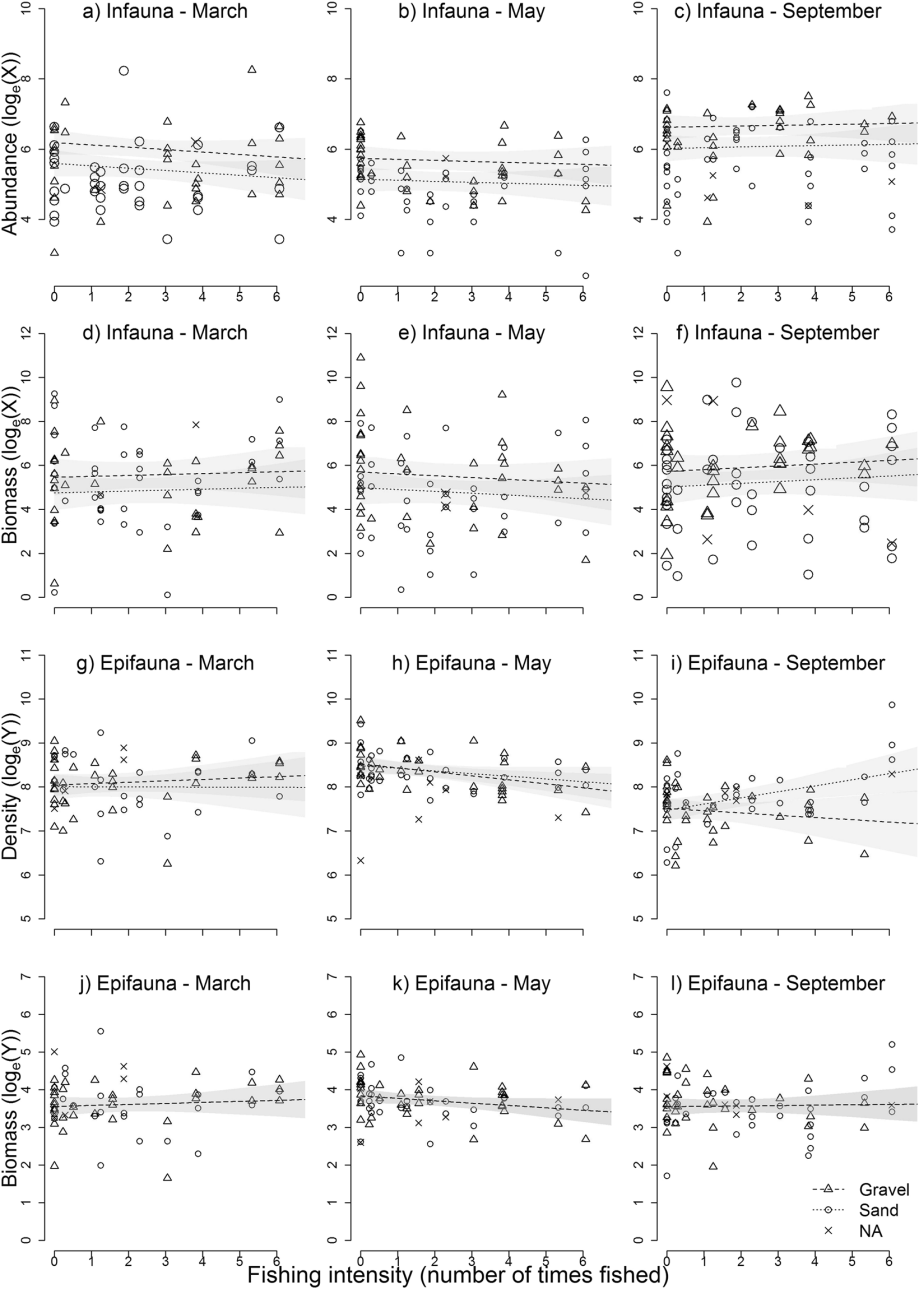
Effect of scallop dredging on total community infaunal (**a–f**) and epifaunal (**g–l**) abundance and biomass (excluding *P. maximus* and *O. fragilis* from the epifaunal analysis). Left to right: March (prior to fishing), May (after fishing) and September (four months after fishing). X is for infauna, measured in number of individuals (x10 + 1) or weight in grams (x100 + 1) per 0.1 m^2^. Y is for epifauna measured in density of individuals (x100 + 1) or weight in kgs (x100 + 1) per 100 m^2^. Each symbol is a sample, distinguishing sand and gravel types, and NA for samples where no sediment information were available (i.e. data not included in the models). Lines show the predicted values from best models with 95% confidence intervals (grey shading) (all models included fishing intensity, survey time and their interaction and other fixed effects as specified in Table 2).

There was a high spatial and temporal variability among samples (Table 2, Fig. 2). Total abundance and biomass of infauna did not change significantly with the fishing intensity (FI) gradient (non-significant Survey time * FI interactions, Table 2). In contrast, the abundance of epifauna in gravel and its biomass in both sand and gravel decreased along the FI gradient directly after the fishing impact (−11% and −9% per dredge pass respectively), but had recovered after four months (Table 2).

**Table 2:** Results of the GLMMs for the BACI experiment on infaunal and epifaunal abundance (Ab) and biomass (Bio) at the population and class or phylum level, for a continuous gradient of fishing intensity (FI).

Response		Fixed effects	ΔAIC	FI x Survey interaction						Survey effect					
				May			September			May			September		
				ES (%) (β_3a(7a)_)	SE_3a(7a)_	p_3a(7a)_	ES (%) (β_3b(7b)_)	SE_3b(7b)_	p_3b(7b)_	ES (%) (β_2a_)	SE_2a_	p_2a_	ES (%) (β_2b_)	SE_2b_	p_2b_
All infauna	(Ab)	T,D,DxS	2.9	4	0.081	0.621	9	0.083	0.294	−35*	0.244	0.075	56*	0.246	0.071
	(Bio)	T	2.59	−12	0.172	0.476	4	0.176	0.808	27	0.529	0.658	30	0.533	0.625
Bivalvia	(Ab)	D,DxS	1.99	10	0.14	0.494	25	0.159	0.16	27	0.441	0.585	89	0.455	0.163
	(Bio)		2.88	−7	0.342	0.842	32	0.345	0.429	14	1.02	0.9	65	1.024	0.627
Crustacea	(Ab)	D,DxS	**−2.66**	19	0.126	0.175	41*	0.133	0.01	−56*	0.397	0.038	170*	0.403	0.014
	(Bio)	T,D	1.95	−17	0.138	0.185	−1	0.151	0.93	124*	0.429	0.061	244*	0.446	0.006
Echinodermata	(Ab)		−**2.11**	−34*	0.175	0.019	−28*	0.197	0.095	125	0.572	0.157	267*	0.604	0.032
	(Bio)	T,D	3.19	−8	0.147	0.579	−13	0.151	0.376	−7	0.377	0.842	28	0.377	0.521
Polychaetae	(Ab)	T,D,DxS	3.13	2	0.096	0.81	9	0.098	0.373	−35	0.29	0.134	44	0.29	0.206
	(Bio)	T,D	−**2.44**	−10	0.1	0.283	15	0.104	0.17	25	0.308	0.467	32	0.314	0.375
Sipuncula	(Ab)		3.12	−12	0.282	0.663	15	0.315	0.654	−35	0.867	0.623	113	0.917	0.41
	(Bio)	T,TxSxFI	5.12	sd: −14	0.139	0.1	−15*	0.096	0.089	5	0.172	0.774	41*	0.172	0.048
				gv: 4	0.077	0.616	5	0.089	0.599						
All epifauna	(Ab)	T,TxSxFI	−**10.77**	sd: −6	0.064	0.369	15*	0.071	0.049	61*	0.133	<0.001	−41*	0.155	0.001
				gv: −11*	0.057	0.034	−8	0.79	0.296						
	(Bio)		0.31	−9*	0.054	0.099	−2	0.061	0.789	32*	0.147	0.061	−1	0.164	0.969
Bivalvia	(Ab)	D	2.52	19	0.159	0.268	18	0.163	0.298	−15	0.43	0.715	−80*	0.438	<0.001
	(Bio)	D, DxS	−**9.54**	32*	0.079	0.001	12	0.081	0.158	−3	0.208	0.884	−32*	0.209	0.062
Bryozoa	(Bio)		−2.05	−8*	0.042	0.038	−10*	0.049	0.026	14	0.115	0.265	24*	0.13	0.098
Chordata	(Ab)	D	0.56	−8	0.061	0.161	2	0.063	0.727	0	0.165	1	−40*	0.165	0.002
	(Bio)	T	−**5.47**	−17*	0.061	0.002	−5	0.062	0.393	93*	0.221	0.003	−10	0.222	0.649
Cnidaria	(Ab)		−**8.17**	−16*	0.072	0.014	−22*	0.073	0.001	69*	0.197	0.008	9	0.2	0.655
	(Bio)		−**8.25**	−18*	0.069	0.005	−21*	0.071	0.001	55*	0.187	0.021	45*	0.187	0.049
Crustacea	(Ab)		−**3.15**	−11*	0.064	0.06	4	0.066	0.507	20	0.177	0.295	−54*	0.178	<0.001
	(Bio)		1.3	−9	0.075	0.234	2	0.077	0.763	1	0.205	0.95	−22	0.207	0.228
Echinodermata	(Ab)	T,D	−**2.98**	−4	0.097	0.693	25*	0.101	0.027	91*	0.265	0.015	−45*	0.265	0.023
	(Bio)	T	−**3.85**	−2	0.08	0.813	20*	0.086	0.033	25	0.219	0.303	−9	0.229	0.685
Gastropoda	(Ab)	D,DxS	2.13	−10	0.101	0.287	2	0.102	0.845	64*	0.264	0.061	−76*	0.276	<0.001
	(Bio)	D,DxS	2.58	−10	0.112	0.344	2	0.113	0.893	35	0.295	0.315	−36	0.296	0.143

S = Survey time, T = Texture, D = Depth. All models include the fixed effects S, FI and SxFI, plus the effects indicated in the table. ΔAIC is the difference in AIC between models with and without the ‘time x treatment’ interaction (i.e. SxFI) -a negative value (bold) means that the model with interaction is better (i.e. significant effect of the fishing gradient). β_3a(7a)_ and β_3b(7b)_ are the estimates of the ‘S x FI’ interaction as in eq1 for ‘before’ to ‘after’ and ‘before’ to ‘4 months after fishing’ respectively, with corresponding standard errors (SE) and p-values (p). β_2a_ and β_2b_ are the estimates (with s.e. and p-values) of the survey effect alone (i.e. natural variation in time). Here we give ES, i.e. effect size equal to [exp(β)-1] * 100, instead of β, for interpretability. ES is the percentage change (in abundance or biomass) per dredge pass compared to March. ‘gv’ and ‘sd’ refer to gravel and sand where the interaction with texture was in the best model. The symbol * identifies the significant ES with α = 0.1. *P. maximus* and *O. fragilis* were excluded from the epifaunal groups.

### Impact of fishing gradient at the taxonomic level

**Figure 3:**
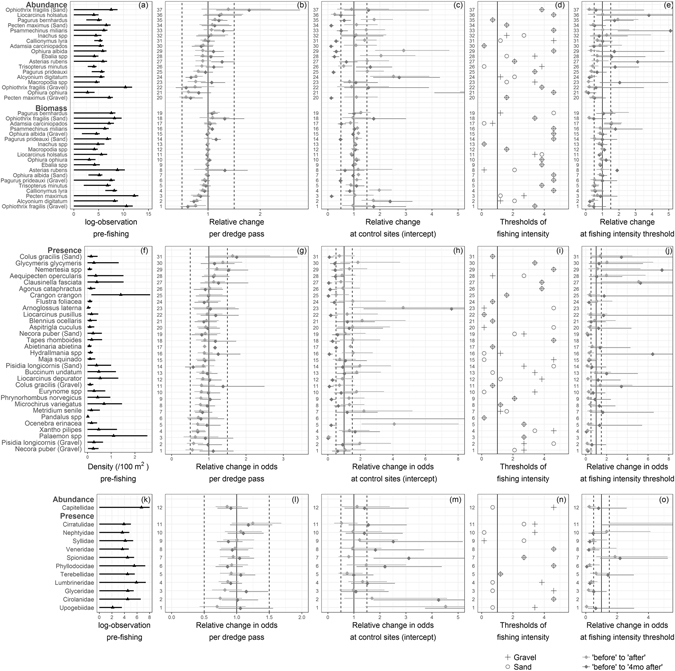
Comparison of the effect size of fishing intensity (FI) vs natural time variation on the abundance and biomass of epifaunal taxa (top row, **a–e**), the presence of epifaunal taxa (midle row, **f–j**) and the abundance and presence of infaunal taxa (bottom row, **k–o**). Some taxa have a different response in sand and gravel as indicated. For each row, the first panel (**a,f,k**) is the pre-fishing observed estimate ± standard deviation (density is given for data analyzed as presence/absence only); densities and biomasses are log-transformed in the top and bottom panels using log(x100), with original data in number or g/100 m^2^. The second panels (**b,g,l**) are the relative changes due to the interaction between FI and survey time and the third panels (**c,h,m**) the survey effect alone, both from the GLMM outputs using continuous FI in the predictor variables. The fourth panels (**d,i,n**) are the taxon-specific FI thresholds and the fifth panels (**e,j,o**) the relative change at those thresholds as estimated from the GLMMs outputs using the categorized FI. Note that for presence this is the change in odds (i.e probability of presence compared to probability of absence). Light grey = before to after fishing (March to May), dark grey = before to four months after fishing (March to September). Estimates shown in the second, third and fifth panels are the effect sizes, exp($\beta$) from eq1, with 90% confidence interval (α = 0.1). Abundance was modeled with Poisson or negative binomial, biomass with gamma distributions and presence with binomial (log-log link) distributions GLMMs. The vertical dash lines are ±0.5. 1 means no change, ±0.5 means ±50% abundance, biomass or odds. Missing values in **(e,j,o)** are taxa for which the model failed to converge.

**Figure 4:**
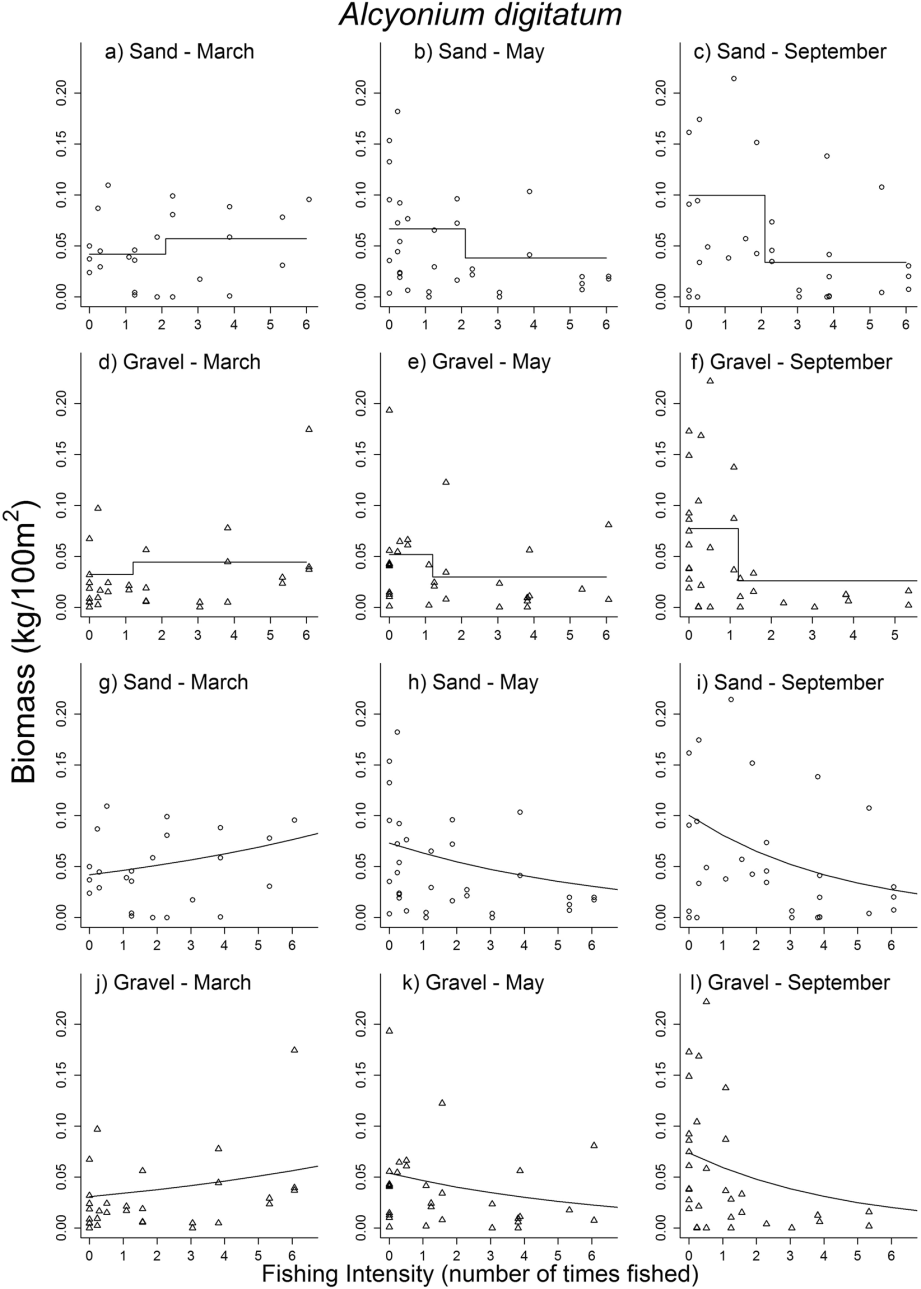
Effect of scallop dredging on the biomass of dead men’s fingers (*Alcyonium digitatum*). Left to right: March (prior to fishing), May (after fishing) and September (four months after fishing). Each symbol is a sample, distinguishing sand and gravel types. Observed values are fitted with GLMMs outputs from the threshold analysis (**a–f**) (best model with AIC = 952.37) and with fishing as a continuous variable (**g–l**) (AIC = 960.13).

The patterns in the responses of different taxa to fishing were more complex than the response of the gross community metrics (see observed data and model fits in Supplementary Figs S2.1 to S2.4). Of the twelve taxonomic groups (class or phylum) tested, five groups had a significant negative response immediately after the FI gradient was implemented, with average decreases between −8% and −18% per dredge pass for four epifaunal groups and −34% for one infaunal group. Cnidarians, bryozoans and infaunal echinoderms remained significantly negatively affected after four months (ca. −21%, −10% and −28% (abundance) per dredge pass respectively) (Table 2). The soft coral dead men’s fingers (DMF, *Alcyonium digitatum*) was the species that accounted most for the response observed in cnidarians, as it constituted 81% and 61% of the cnidarian biomass and abundance respectively (the remainder of cnidarians were mostly the cloak anemone that lives on hermit crabs shells *Adamsia carciniopados* (Supplementary Table S1.2)). DMF biomass and abundance decreased along the FI gradient by 27% and 33% per dredge pass respectively after four months (p-values < 0.001) (Figs 3b and 4). The biomass of bryozoans comprised mostly the hornwrack *Flustra foliacea* (60%) and Ross coral *Pentapora foliacea* (28%). Although the presence of *F. foliacea* did not demonstrate a significant response to fishing (Fig. 3g), these two species accounted most for the negative response of the bryozoan biomass. *P. foliacea* was present in 21% of the hauls before impact and reached up to 50 g/100 m^2^ in one haul but was not modelled here as we set a quality assurance threshold of 25% presence in samples for a taxon to be considered for modelling. Unlike cnidarians, bryozoans and infaunal echinoderms, the abundance of epifaunal crustaceans and the biomass of chordates (fish), which were also initially negatively impacted by the fishing gradient, appeared to have recovered after four months (Table 2 and Supplementary Figs. S2.1 and S2.2). This was illustrated by some of the dominant species (e.g. the fish *Callionymus lyra* or *Trisopterus minutus* and the small crabs *Macropodia* spp. or *Pagurus prideauxi*) (Fig. 3b).

**Table 3:** Results of the GLMMs for the BACI experiment on infaunal and epifaunal abundance and biomass at the population and class or phylum level for categorized fishing intensity (FI), i.e. threshold analysis.

Response		Fixed effects	ΔAIC	Threshold	Categorical FI x Survey interaction					
					May			September		
					ES (%) (β_3a_)	SE_3a_	p_3a_	ES (%)(β_3b_)	SE_3b_	p_3b_
All infauna	(Ab)	D, DxS	**−1.83**	0.7	−20	0.357	0.527	87*	0.362	0.083
	(Bio)	T, D	0.34	4.6	−71	0.837	0.139	−79*	0.899	0.081
Bivalvia	(Ab)	D, DxS	**−0.35**	gv:1.6; sd:1.2	20	0.655	0.777	230*	0.653	0.067
	(Bio)	T	**−1.86**	1.6	0	1.143	0.999	1148*	1.172	0.039
Crustacea	(Ab)	D,DxS	**−4.67**	2.1	117	0.555	0.162	423*	0.557	0.003
	(Bio)	T,D	**−2.7**	gv:1.2; sd:0.7	−70*	0.658	0.068	81	0.67	0.379
Echinodermata	(Ab)		**−2.94**	4.6	−90*	1.092	0.036	−93*	1.126	0.018
	(Bio)	T,D	**−6.02**	4.6	−15	1.019	0.869	−86*	0.899	0.032
Polychaetae	(Ab)	D,DxS	**−0.34**	0.7	−30	0.428	0.411	74	0.433	0.201
	(Bio)	D	**−6.71**	2.7	−28	0.426	0.447	171*	0.429	0.021
Sipuncula	(Ab)		1.01	gv:1.2; sd:0.15	−81	1.279	0.189	41	1.268	0.788
	(Bio)	T	2.96	3.85	12	0.635	0.856	−39	0.658	0.459
All epifauna	(Ab)		**−13.07**	gv:3.4; sd:4.6	−52*	0.18	<0.001	−2	0.352	0.96
	(Bio)	D	**−5.93**	4.6	−56*	0.239	0.001	−11	0.342	0.739
Bivalvia	(Ab)		**−6.66**	3.85	344*	0.647	0.021	833*	0.654	0.001
	(Bio)	D	**−17.24**	2.7	293*	0.284	<0.001	79*	0.322	0.072
Bryozoa	(Bio)		**−3.01**	2.1	−36*	0.177	0.014	−39*	0.191	0.012
Chordata	(Ab)	D	**−1.37**	4.6	−47*	0.35	0.071	17	0.365	0.663
	(Bio)	T	**−6.04**	4.6	−63*	0.277	0.001	−16	0.272	0.514
Cnidaria	(Ab)		**−11.46**	gv:1.2; sd:2.1	−50*	0.296	0.021	−70*	0.302	<0.001
	(Bio)		**−7.09**	gv:1.2; sd:2.7	−49*	0.26	0.009	−62*	0.302	0.002
Crustacea	(Ab)		**−2.16**	1.6	−37*	0.275	0.088	24	0.277	0.444
	(Bio)		**−4.35**	0.15	−46*	0.299	0.039	20	0.299	0.542
Echinodermata	(Ab)	T	**−3.02**	gv:3.85; sd:4.6	−36	0.501	0.37	144*	0.532	0.094
	(Bio)	T	**−7.17**	2.1	9	0.299	0.775	145*	0.301	0.003
Gastropoda	(Ab)	T,D	**−3.07**	gv:3.4; sd:4.6	−56*	0.485	0.089	60	0.531	0.377
	(Bio)	T,D	1.51	gv:0.15; sd:1.6	−43	0.39	0.155	−40	0.396	0.205

Threshold is the FI that split low FI from high FI. β_3a_ and β_3b_ are the estimates of the ‘S x FI’ interaction as in eq. 1 for before to after and before to four months after fishing respectively, with corresponding standard errors (SE) and p-values (p). βs are expressed as percentage change, i.e. effect size (ES) (See caption Table 2). Here, ES is the percentage change at the FI threshold. *P. maximus *and *O. fragilis *were excluded from the epifaunal groups.

Some taxa had positive changes post-fishing relative to unfished areas. At the class level, only the biomass of epifaunal bivalves increased along the FI gradient directly after fishing (+32% per dredge pass), but this change was not significant after four months (Table 2). Several species might have contributed to this pattern owing to their presence/absence (e.g., *Glycymeris glycymeris, Aequipecten opercularis* and *Clausinella fasciata*) (Fig. 3g). The overall positive increase in epifaunal abundance along the FI gradient four months after impact was attributed to significant increases in epifaunal echinoderms (+25% per dredge pass), such as starfish *Asterias rubens* and the sea urchin *Psammechinus miliaris* (Fig. 3b). Overall infaunal abundance also tended to increase along the FI gradient after four months (although not significantly) due to a significant increase in infaunal crustaceans of 41% per dredge pass which was not attributed to any individual family taxon (Fig. 3l).

### Effect on the target species

The impact of fishing on the target species provided further insights into the power of our experimental design to detect changes in the abundance of species. Fishing had the strongest negative direct impact on the abundance the target species of *P. maximus* in gravel (−42% per dredge pass, p < 0.001) with some signs of recovery four months later (−33%, p = 0.024) (Fig. 3b). The abundance of *P. maximus* did not change significantly in sand after fishing (+8%, p = 0.339 directly after fishing; +12%, p = 0.371 four months later). In general, fewer scallops were caught in the beam trawl in sand compared to gravel which tends to be their preferred habitat (pre-fishing densities 6.5 scallops/100 m^2^ in sand vs 12.1/100 m^2^ in gravel). The effect on overall scallop biomass was only significant directly after fishing (−18%, p = 0.09 directly after fishing; −14%, p = 0.18 four months later). Our analysis included both undersized and commercial-sized scallops, hence a large fraction of the scallops remaining after fishing will have been undersized scallops, many of which would have not been caught by the fishing vessels or discarded.

### Thresholds analysis and natural variation

The threshold approach, which used a categorized FI, identified more significant interactions between fishing and survey time (25 vs 15 at the phylum/class level) and was generally the preferred approach with respect to AIC, i.e. more parsimonious models than those with the continuous FI gradient (Table 4). This was illustrated by DMF (ΔAIC = −7.76) (Fig. 4) (see also Supplementary Figs S2.1 to S2.4 for all the model fits at higher taxonomic levels). As for the continuous FI gradient, the effect of the categorized FI was predominantly negative directly after impact (except for epifaunal bivalves), followed by a mix of positive and negative responses four months later (Table 3, Supplementary Fig. S2.5).

**Table 4:** Comparison of detection power of models with continuous or categorized (threshold) fishing intensity (FI) as a predictor variable.

**Response**		**ΔAIC**	**Model with continuous FI**				**Model with categorical FI (threshold)**			
			**Power to detect a change of**				**Power to detect a change of**			
			**15%**		**25%**		**25%**		**50%**	
			**mean**	**95%ci**	**mean**	**95%ci**	**mean**	**95%ci**	**mean**	**95%ci**
All infauna	(Ab)	9.91	65	58–71	**97**	**93–98**	21	15–27	58	51–65
	(Bio)	1.57	23	18–29	45	38–51	9	6–14	9	5–13
Bivalvia	(Ab)	2.73	32	26–39	69	62–75	19	14–25	38	31–44
	(Bio)	4.33	14	9–19	25	19–31	7	4–11	7	4–11
Crustacea	(Ab)	1.38	30	24–37	66	59–72	18	13–23	32	26–39
	(Bio)	8.19	41	34–47	78	**71–83**	19	14–25	45	38–51
Echinodermata	(Ab)	2.38	21	15–27	45	38–52	19	14–25	19	14–24
	(Bio)	7.55	42	35–49	78	**72–83**	17	12–22	33	26–39
Polychaetae	(Ab)	−34.57	64	57–70	**95**	**90–97**	22	16–28	55	48–61
	(Bio)	3.73	40	33–47	76	**70–81**	15	11–21	44	37–51
Sipuncula	(Ab)	2.97	21	16–27	32	26–39	17	12–22	20	15–26
	(Bio)	55.2	38	32–45	79	**73–84**	15	10–20	63	56–69
			27	21–34	58	51–65				
All epifauna*	(Ab)	4.1	**94**	**90–97**	**100**	**98–100**	27	21–34	73	66–79
			**97**	**93–99**	**100**	**98–100**				
	(Bio)		**87**	**82–91**	**100**	**98–100**	21	16–27	53	46–59
Bivalvia*	(Ab)	6.7	40	33–46	60	53–66	18	13–24	28	22–34
	(Bio)	8.11	63	56–69	**96**	**92–98**	14	9–19	14	9–19
Bryozoa	(Bio)	22.51	**97**	**93–99**	**100**	**98–100**	68	61–74	**100**	**98–100**
Chordata	(Ab)	1.52	**84**	**78–88**	**100**	**98–100**	28	22–34	60	53–66
	(Bio)	8.61	61	54–67	**96**	**92–98**	13	9–18	36	29–42
Cnidaria	(Ab)	7.26	72	65–77	**99**	**96–100**	26	20–33	**81**	**75–86**
	(Bio)	11.31	71	64–77	**99**	**96–100**	27	21–33	**81**	**75–86**
Crustacea	(Ab)	2.6	**83**	**77–87**	**100**	**98–100**	32	26–39	**82**	**76–87**
	(Bio)	7.61	76	**69–81**	**100**	**98–100**	20	15–26	61	54–68
Echinodermata*	(Ab)	8.49	58	51–64	**94**	**90–97**	17	12–23	48	41–54
	(Bio)	9.09	62	55–68	**96**	**92–98**	26	20–32	64	57–70
Gastropoda	(Ab)	3.28	54	47–61	**85**	**79–89**	16	12–22	50	43–57
	(Bio)	35.23	24	18–30	61	54–67	11	7–16	40	33–47

ΔAIC is the difference in AIC between models using continuous FI as a predictor versus the threshold approach. A positive value means that the best model was the threshold model. The power to detect a decrease of 15% and 25% per dredge pass with the continuous FI approach is reported for α = 0.1, as well as the power to detect a decrease of 25% and 50% with the threshold approach. The values in bold are the values which exceed a power of 80%.

The identified threshold values varied per taxon or group of species suggesting a mosaic of individual responses to fishing (Table 3, Fig. 3). At the taxon level, taxa that underwent a major change in abundance or presence at a fishing intensity threshold of <2.5 times fished in sand tended to show a negative response to fishing, directly after impact (Supplementary Fig. S2.5a). Above that threshold more resilient taxa displayed a mix of positive and negative responses. This pattern was not as pronounced in gravel (Supplementary Fig. S2.5b). On average, in sand, changes occurred at lower fishing intensity thresholds than in gravel (Fig. 3, Supplementary Fig. S2.5e,f). After four months, there was no clear threshold value affecting the benthic community as a whole.

The threshold analyses suggested some non-linear patterns of similar or relatively smaller amplitude than the natural variability gradient (Fig. 3, compare third and fifth panels respectively showing natural variation and threshold effects (Fig. 3c/e,h/j,m/o), see also Supplementary Fig. S2.6 for visual comparison of point estimates of effect sizes). From the models using the continuous FI gradient, fishing an area >6 times (maximum FI in the range of this study) was a requisite to match the level of overall natural variability observed at the community level after 4 months (Supplementary Fig. S2.6).

### Power analysis

The effect sizes (ES) detected at the group (class or phylum) level with the continuous FI approach varied between −34% and +41% per dredge pass with the smallest negative and positive ES detected being −8% and +15% (Table 2). With the threshold approach, the ES were as expected more dramatic with a range of −90% to > + 1000% (total, as opposed to per dredge pass) (Table 3). The minimum negative and positive ES detected were −36% and +79%. The power of the experiment to detect a decrease of 25% per dredge-pass with the continuous FI gradient approach was above 80% for all epifaunal groups for either abundance, biomass or both (Table 4), with enough power to detect changes as low as 15% change for bryozoans, chordates and crustaceans. A decrease of 25% in infauna could be detected with a power close to 80% for most groups. Using the threshold approach, the effect had to be large (>50%) to be detected at the group level (except for epifaunal crustacean, bryozoan and cnidarians). These patterns were also true at the species (or family) level despite a lower power on average (Supplementary Fig. S2.7).

### Community Composition Analysis

Despite a lower turnover rate overall, seasonal changes in epifauna were more pronounced than infaunal ones (see BC indices model estimates Table 5). A post-hoc test showed no significant difference between the March-May and the March-September dissimilarity estimates for infauna (p = 0.726) and a significant one for epifauna (p < 0.001). However, the level of dissimilarity in epifaunal community composition from before to after fishing remained constant along the FI gradient (no significant interaction between FI and surveys, Table 5), in spite of the taxa specific responses reported earlier. In contrast, the infaunal community composition dissimilarity from pre-fishing conditions did gradually increase along the FI gradient directly after impact but the response was no longer observed after four months. The spatial variation along the fishing gradient (within survey) mirrored the temporal variation, i.e. only infauna showed a significant change in composition along the fishing gradient but the short-term effect was no longer significant after 4 months (Table 5). Note that the analyses presented here were conducted on abundance data but that tests were also conducted on biomass data for epifauna (not presented here) and did show an increase in dissimilarity along the gradient in May which was no longer significant in September (much as for infauna).

**Table 5:** Effect of fishing intensity (FI) on Bray Curtis community dissimilarity estimates, assessed with linear mixed effect models.

Model	Community	Variable	Estimate	SE	p-value
Temporal	Infauna	March-May Surveys	0.742	0.009	<0.001
		March-September Surveys	0.780	0.009	<0.001
		FI gradient	0.001	0.004	0.828
		FI x March-May Surveys	0.015	0.003	<0.001
		FI x March-September Surveys	0.003	0.003	0.344
	Epifauna	March-May Surveys	0.484	0.022	<0.001
		March-September Surveys	0.603	0.022	<0.001
		FI gradient	0	0.006	1
		FI x March-May Surveys	0.004	0.008	0.629
		FI x March-September Surveys	−0.004	0.008	0.647
Spatial	Infauna	March Control-Fished	0.789	0.01	<0.001
		May Control-Fished	0.744	0.01	<0.001
		September Control-Fished	0.712	0.01	<0.001
		FI gradient	0	0.002	1
		FI x March Control-Fished	0.002	0.003	0.533
		FI x May Control-Fished	0.011	0.003	<0.001
		FI x September Control-Fished	−0.001	0.003	0.595
	Epifauna	March Control-Fished	0.518	0.03	<0.001
		May Control-Fished	0.471	0.03	<0.001
		September Control-Fished	0.583	0.03	<0.001
		FI gradient	0	0.007	1
		FI x March Control-Fished	0.003	0.01	0.759
		FI x May Control-Fished	0.008	0.01	0.418
		FI x September Control-Fished	0.008	0.01	0.412

The temporal models compared the within-site differences between surveys, i.e. seasonal turnover, while the spatial models compared the within-survey differences between control and fished sites, i.e. spatial turnover. Given are model estimates of BC indices, standard errors (SE) and p-values.

## Discussion

The present BACI experiment, to our knowledge, was the largest controlled dredging impact experiment conducted to date in terms of the large spatial extent of the seabed manipulated and in terms of the range of fishing intensities studied. The power to detect the effects of fishing on whole communities and at the phylum or class and family levels was high^22–26^. Most of the studies summarized in Supplementary Table S2.2 could detect large (i.e. >50% change) effects of fishing mortality. In contrast, the present study could detect effect sizes at the class or phylum level of 25% to 15% for some epifaunal groups.

The overall effect of fishing in the SAC was found to be modest and short-lived for the majority of taxa. The short-term seasonal variation in the benthic fauna in this area was large, and exceeded that of the effect of fishing for most taxonomic groups, concurring with Sciberras *et al*.^21^. A comparable smaller-scale study conducted by LeBlanc *et al*.^25^ estimated that the magnitude of short-term natural changes in fauna in an unconsolidated seabed area of the NW Atlantic, Canada, was similar in magnitude to that produced at intensively fished sites (i.e. fished 10 times). In the present study, the natural temporal fluctuations at control sites suggested that a fishing disturbance equivalent to >6 times fished is closest to the magnitude of seasonal changes that occur between March and September in Cardigan Bay. Benthic faunal composition can be expected to change significantly over the summer months, as observed here, due to processes such as spring recruitment, growth or migrations. These changes may have impaired the detectability of the responses to fishing impact by increasing the variability of the dataset. However, since control sites were used as a baseline for each survey, this was unlikely to be a significant limitation of the study. Further, if temporal variability hid the effect of fishing, this implies that natural changes were greater in magnitude than the changes caused directly by fishing.

In the present study the small effect of fishing relative to seasonal variations is likely to be related to the high levels of natural disturbance of the seabed by waves in the area, leading to an impoverished and highly variable benthic fauna associated with an unstable and mobile substratum^21^. Natural disturbance exceeds disturbance generated by fishing effects in many inshore areas of the seabed around the UK^13, 14^ and has a similar effect on the benthos as fishing^15^. In Cardigan Bay the underlying gravels and cobbles are overlain by a veneer of highly mobile sands with large-scale changes in topography^21^. Our findings are further corroborated when placed in the context of previous local and UK wide studies^27, 28^. Kaiser and Spencer^27^ undertook a spring survey in North Wales and reported an average of 28.5 infaunal species and 58.5 individuals/0.1 m^2^ in a mobile sandy seabed that was unfished (similar to this study, assuming two infaunal species per family based on previous unpublished data collected in Cardigan Bay in October 2012) vs 66.8 species and 334.8 individuals 0.1 m^−2^ in more stable areas. Bolam *et al*.^28^ places Cardigan Bay in the 23rd percentile at the UK scale in terms of infauna biomass, 25th for abundance and 35th for species richness as well as being in the 43rd percentile for tidal stress and 76th for peak wave stress.

Although the effect of fishing at the population level was small and/or of short duration, significant and enduring effects of fishing were found for some specific taxa, i.e. Bryozoa and Cnidaria, with reductions of ≥40% (past a given threshold) in abundance and biomass persisting four months after fishing. Both these groups consist of sessile, emergent epifauna that are likely to be dislodged or crushed by a passing scallop dredge^9, 29^. Much of the effect on these groups seems to be related to effects on the soft coral *A. digitatum* and possibly Ross coral *P. foliacea*. *A. digitatum* contributes greatly to the benthos wet weight biomass (5^th^ most important species, Supplementary Table S1.2). *P. foliacea* is a large brittle bryozoa which was only observed in sites fished ≤1.9 times directly after fishing and four months later, despite having been observed pre-fishing in four sites that were going to be fished between 2.3 and 6.1 times. Their recovery from fishing may take >5 years^11^, although signs of recovery were observed for both these species in Lyme Bay, UK, within three years after the area was closed to scallop dredging^30^. If management of scallop fisheries was targeted at the protection of the most sensitive groups rather than the overall abundance and biomass of fauna in the ecosystem, our threshold analysis suggests that the dredging intensity should not exceed one pass on gravel and two passes on sand due to its effect on *A. digitatum* and apparent lack of recovery after four months. It has to be noted that the study was not designed specifically to separate the effects of fishing on sand and gravel, i.e. the fishing boxes were composed of both types of sediment as the experimental area was patchy, and so the conclusions with respect to sediment type must be interpreted with caution. If the benthic community was to be monitored as a whole then the area could be fished up to 6 times before changes of the same level as the natural variation occurred. However, the study being a BACI experiment, the chronic effect of fishing would have to be continuously monitored to insure that there would be no long-term trend in the taxa-specific changes that we observed after four months and that could affect the population structure (i.e. decrease in sessile organisms and increase in scavengers).

Our study suggests that, overall, recovery occurred in four months, which is fast in comparison to other larger scale studies of real fishing activities (as opposed to BACI experiments). It would have been preferable to sample several times more over the following years. However, even on heavily scallop dredged grounds in the Irish Sea some communities commence rapid recovery during the first year post-fishing disturbance^12^, and there is no reason to assume this would be different in Cardigan Bay, especially in the context of high energy environment that predominates the biology at this location. The reason for resampling after four months was mostly logistical but it also corresponded approximately to the duration of the current closed season (six months over summer) and therefore made sense in order to study potential management options for the SAC in light of the current management system in place.

Our results are also comparable to those of other published large-scale BACI studies conducted in habitats with similar high energy regimes that reported natural variability to exceed fishing effects on the benthos^22, 23, 25, 31–33^. Complex responses were observed a few months after the initial impact but, generally, some functional characteristics of the taxa are discussed as important factors driving the response to fishing such as feeding type, depth of occurrence of infauna, size or brittleness of epifauna. These patterns seem to be common in sandy-gravelly type of fishing grounds^23, 26, 34^. For instance the counter-intuitive initial positive response of some bivalves species, i.e. increase in abundance or biomass along the fishing gradient, was also observed in other studies^23, 32^. Explanations for this response include the potential for a change in catchability due to the reworking of sediment by the dredges or some post-disturbance settlement event. Another non-instinctive finding was the lack of significance of the effect of dredging on the target species in sand. Since the size of the scallops caught in the beam trawls was not recorded, it cannot be ruled out that a significant amount of scallops in sand could have been undersized and therefore returned to the seabed during fishing operations. However a driving factor is likely to be that the vulnerability of scallops to the sampling gear is different in sand and in gravel as scallops may bury deeper in sand and may therefore not be sampled consistently.

A criticism of BACI and other *in-situ* experiments is that they are small-scale compared to the impact created across an entire fishing ground^10, 35^. The recovery in areas disturbed at the scale of a fishing ground will have different dynamics because reproduction and growth may be the main source of observed recovery if immigration from surrounding areas is limited. In contrast, active migration may have a more important role in recovery dynamics for patches of small-scale disturbance^12, 36^. Here, we have attempted to overcome the limitations of previous studies by fishing across large areas of the seabed. Nevertheless, a large proportion of the scallop ground around the experimental sites remained unfished and thus undoubtedly could provide an adjacent source of breeding adult fauna providing a source of recruits and mobile individuals capable of active immigration.

Considering the relatively high value of the landings from a relatively small area of the seabed (the value at first sale of scallops caught during this experiment was equivalent to ca. USD 500 000 in April 2014), these observations have interesting implications for sustainable management of bottom trawl and dredge fisheries. Targeted harvesting of smaller areas of the seabed could result in more rapid post-fishing recovery dynamics if surrounding areas of the seabed remain unfished or are fished at lower intensities^12^. Such an approach would increase economic efficiency and minimise impacts on species of conservation concern.

Detecting the effects of fishing disturbance is difficult when fishing occurs in areas of the seabed subject to high natural disturbance that naturally support benthic communities with low benthic diversity, abundance and biomass (see Gray *et al*.^37^ for a discussion on this issue). It is often advocated that management should take a precautionary approach, where avoiding possible negative effects on the environment is a high priority even when this could result in unnecessarily restricting fishing activity. In such a framework, avoiding Type II errors is equally important as avoiding Type I errors. A Type II error is the probability of missing a significant impact by accepting the null hypothesis. We minimized Type II errors by using α = 0.10 and by not applying corrections for multiple testing, but as a result, it is very likely that some of our significant results are Type I errors (in particular some of the positive effects of fishing) and results need to be interpreted with care. Nevertheless, we considered that given the conservation importance of Cardigan Bay SAC it was appropriate to err on the side of extreme caution.

Current advances in vessel monitoring systems and fishing gear monitoring systems mean that it is now feasible to monitor in real time the amount of seabed disturbance that occurs and to consider the development of management systems that operate with disturbance or impact management trigger points^38^. Such an approach to fisheries management could be considered to move towards an example of best-practice for managed towed-bottom gear fisheries.

## Methods

### Experimental Design

The impact of fishing was examined across a gradient of scallop dredging intensities with experimental sites spread over a 110 km^2^ area (approximately 8 km by 13.5 km) located between 3 and 12 nautical miles (nm) offshore (Fig. 1). Previous surveys indicated that the seabed was relatively flat and homogeneous in this area and consisting mostly of muddy gravel, sand and broken shells. The experimental layout consisted of four control sites in which no fishing occurred and 13 impact sites each of which was exposed to a different fishing intensity. The distribution of the fishing intensities, including control sites, was randomized in space among these 17 sites (Table 1). Fishing intensity was defined as the number of times an area was fished entirely (i.e. every m^2^ covered by a dredge). Thus if a fishing vessel swept 1.2 km^2^ of seabed concentrated in a pre-determined area of 0.63km^2^, it would have fished the area on average 1.2/0.63 = 1.9 times. We achieved a gradient of fishing that ranged from 0 (controls) to a maximum intensity of 6.1 times fished.

Each fishing vessel fished with Newhaven dredges which are 0.76 m wide and have 12 cm long teeth. Each fishing site comprised three zones: one fishing zone of ca. 1700 m long by 370 m wide, and two zones at either end in which the vessels were allowed to manoeuvre (haul, shoot and turn) (Fig. 1). The aim was to spread the tows homogeneously across the width of each site. The catch was processed as per commercial practices, with legal-sized scallops retained (>110 mm wide) while undersized scallops and by-catch were discarded over the fished area.

### Data collection

Each site was sampled prior to fishing in March 2014, directly post-fishing in May 2014, and again four months later in September 2014. A minimum of 72 h was left after the last fishing event to allow for damaged organisms to die and for the predators and scavengers to feed on them before sampling^36^. The last survey, in September, was conducted in the early autumn as recovery is expected to be fastest over the summer due to warmer water temperatures and summer recruitment. Previous studies have shown that recovery of benthic ecosystems from bottom fishing can take many years to occur, and hence we did not assume *a priori* that full recovery would occur within four months in our analyses.

### Infauna and sediment sampling

Five to nine 0.1 m^2^ Hamon grab samples were taken at each site on each sampling occasion. The samples were spread out at random inside each fishing area. A sediment subsample (ca. 40 g) was taken from each sample and frozen for particle size analysis (PSA) following standard techniques^39^ and categorized according to Folk^40^ and grouped into ‘gravelly sand’ and ‘sandy gravel’ (including different sub-groups with various proportions of mud content). Hereafter we refer to these sediments as ‘sand’ (gravelly sand) and ‘gravel’ (sandy gravel) for simplicity (Table 1).

The fauna was sieved over a 1 mm sieve and analyzed in compliance with national quality assurance guidelines^41^. All animals were identified to family level whenever possible and counted. Colonial organisms were recorded as present and counted as 1 in abundance data analyses. All animals were then aggregated into 20 predefined taxonomic groups (of class, subphylum or phylum) prior to being weighed (blotted wet-weight) and an estimate of biomass per group was produced.

### Epifauna sampling

During each survey, three to five tows (each 5 minutes in duration) were conducted with a 2m-beam trawl (fitted with a 4 mm mesh net) across the width of each of the 17 sites at a speed of ca. 1.8 knots, resulting in approximately 275 m long tows. The samples were spread out inside each plot to ensure homogeneous coverage. The fauna was sorted on deck and all individuals identified to the lowest taxonomic level possible and weighed wet at that level (±1 g). Colonial taxa such as hydroids were recorded as present and weighed.

### Data analyses

All analyses were conducted separately on infauna (grab samples) and epifauna (beam trawl samples). Abundance and biomass data were analyzed as total abundance and total biomass, i.e. all faunal data were combined per sample. Scallops *Pecten maximus* and brittlestars *Ophiothrix fragilis* were removed from the total epifauna and analyzed separately because scallops were the target species of the fishery (i.e. they were consistently removed from the seabed) and brittlestars accounted for a disproportionately large percentage of total abundance and would mask any effects on other groups. Data were further aggregated within taxonomic groups (class to phylum) such as bivalves, echinoderms, gastropods, cnidarians, bryozoans, polychaetes or chordates (fish) or analyzed at lower taxonomic levels. Meiofauna (i.e. copepods and nematodes) and mysids (ephemeral and mostly pelagic organism) were removed from infauna data analyses.

### Abundance and biomass

Samples taken within sites were not true replicates, this was accounted for by using mixed effect models. Generalized linear mixed models (GLMMs) allowed us to correctly specify the structure of the BACI experiment, distinguishing between fixed (fishing intensity (FI) and survey time (S) and their interaction, i.e. the BACI effect) and random effects (site or site-within-survey effect)^42^. The main effect of interest in the GLMMs (full model in eq. 1) was the effect of the interaction of FI and S on abundance and biomass of infaunal and epifaunal data, as this interaction indicates whether fishing had an effect and whether recovery occurred. As the sampling area covered a relatively large and heterogeneous area of the seabed, depth and sediment type (here called ‘texture’) were also included as fixed effects and therefore potential drivers of biomass and abundance. FI and depth were not correlated (Pearson correlation coefficient 0.04, p = 0.542) while both textures were found at most sites (Table 1). The model selection process is described in details below.

1 $${\mu }_{{\rm{ij}}}={{\rm{\beta }}}_{0}+{{\rm{\beta }}}_{1}{{\rm{FI}}}_{{\rm{j}}}+{{\rm{\beta }}}_{2}{{\rm{S}}}_{{\rm{i}}}+{{\rm{\beta }}}_{3}{{\rm{S}}}_{{\rm{i}}}{{\rm{FI}}}_{{\rm{j}}}+{{\rm{\beta }}}_{4}{{\rm{D}}}_{{\rm{ij}}}{{\rm{S}}}_{{\rm{i}}}+{{\rm{\beta }}}_{5}{{\rm{D}}}_{{\rm{ij}}}+{{\rm{\beta }}}_{6}{{\rm{T}}}_{{\rm{ij}}}+{{\rm{\beta }}}_{7}{{\rm{T}}}_{{\rm{ij}}}{{\rm{S}}}_{{\rm{i}}}{{\rm{FI}}}_{{\rm{j}}}+{{\rm{\alpha }}}_{{\rm{j}}}+{{\rm{\varepsilon }}}_{{\rm{ij}}}$$

Where µ_ij_ is the abundance or biomass * i* at site *j*, β_0_ the intercept, FI_j_ the fishing intensity at site *j*, S_i_ the survey corresponding to observation µ_ij_, D_ij_ the depth and T_ij_ the sediment texture at *ij*. βs are the parameters for the predictors, with β_3_ and β_7_ corresponding to the interaction between FI and S (including all three surveys, with the pre-fishing survey as reference point) and therefore being the main parameters of interest of the BACI experiment. β_2_, the survey effect (i.e. 2 parameters, one for the May survey, directly after fishing, and one for the September survey, four months later), was also of interest to compare the magnitude of the fishing effect to natural variation in time. α_j_ are the site-specific effects (or site-within-survey) and ε_ij_ the residuals following a specific exponential family distribution depending on the properties of the modelled response (Table S2.1). The models that used a lognormal residuals distribution were further improved by including a variance component of exponential form that dealt with any heteroscedasticity in relation to a particular variable, providing it reduced the AIC. A stepwise selection procedure using maximum likelihood and based on the Akaike Information Criterion (AIC) was applied to determine the most parsimonious set of predictor variables (fixed and random) for each response variable, constraining the minimum model to include the main effects FI and S and their interaction. The final statistics were estimated using restricted maximum likelihood. Analyses were conducted with R packages *nlme* and *lme4* ^43, 44^. When required, βs were back-transformed using exp(β)-1, unless specified otherwise, to be reported as percentage change from pre- and post-fishing, hereafter referred to as effect size.

Only taxa or groups of species that were present in ≥25% of the samples were analyzed individually to limit the study to non-zero-inflated datasets. We examined abundance and biomass for some taxa but only presence/absence for taxa in low densities (i.e. <4 individuals per 100 m^2^ for beam trawl data - cut-offs determined from data exploration, see Table S2.1). We checked the power of the experiment (for Type II errors) to detect a decrease of 15 and 25% in abundance, biomass or presence per dredge pass was tested by simulation using the R *simR* package for mixed effect models^45^. The specified level of significance was 0.1 for a target power of 80%^23^.

Following previous studies, we increased the level of significance α to 0.1, not to inflate the significance of the results, but to provide conservative management advice in light of the potentially limited power of this type of experiment and, following this same logic, a correction for false discovery rate was not applied here even though a large number of tests were performed on the same dataset^22, 23^. This means that the power to detect negative effects of fishing was maximized, and limits the chance of failing to detect important effects of fishing that need to be taken into account in the management of the fishery. At the same time, it does increase the chance of Type I errors, and it is very likely that some of the significant effects that were detected are purely due to chance.

### Thresholds

We further explored the existence of thresholds, defined as fishing intensity levels that would cause a sudden, significant change in taxon abundance, biomass or presence, that could be used as reference points for management strategies. Here FI was treated as a categorical variable. The approach aimed to define a point at which there was a significant change in the observations between ‘low FI’ and ‘high FI’. This point was chosen to maximize the explanatory power of the model. For each response variable, nine different cut-off points were tested, between 0.15 and 4.6 times fished, by running the univariate model selection described earlier but replacing the continuous FI by a categorical FI (eq. 1). The nine best models were then defined, one for each FI cut-off point, and the model with the lowest AIC was selected for each response variable. The main difference here, compared to the earlier models, was that the models were first run separately for sand and gravel samples in order to define the best cut-off points by sediment type (with T removed from eq. 1). Two models were then compared by AIC, one with a different FI cut-off point per sediment type and another one with a unique FI cut-off point for all sediments. The model with the lowest AIC was the final model and the existence of a threshold was confirmed if the interaction between the categorized FI and survey time was significant in the model. Note that the 3-way interaction between sediment type, FI and survey was not tested here since it was rarely found to be significant in the linear models with a continuous FI. This approach can identify a difference in FI thresholds between sediment types but the effect size is modelled to be the same regardless.

The power of the experiment to detect a decrease of 25 or 50% in abundance, biomass or presence, at the fishing threshold identified for each taxon, was tested by simulation in a similar manner to the models with continuous fishing intensity. Note that the effect size tested is higher here than for the effect size tested in the continuous FI approach as the threshold approach would aim at detecting the largest sudden changes (as opposed to gradual per dredge pass-changes). The fit of the threshold model was compared to the fit of the model with continuous fishing gradient by AIC (models were fit on the same datasets and using the same R functions to ensure consistency and comparability).

### Composition data

Further analyses were undertaken to see whether overall community composition changed across the fishing gradient. Analyses of the changes in taxon composition were all performed on the abundance datasets (square-root transformed) as infaunal biomass was only available at a higher taxonomic level of aggregation. The impact of fishing on the turnover of taxa, or β-diversity, was investigated using the Bray Curtis (BC) dissimilarity index^22, 25, 46^. Two datasets were derived one providing BC indices of temporal variation, i.e. within sites across surveys (March vs May and March vs September for each site), and one providing BC indices of spatial variation, i.e. within surveys across sites (control vs fished for each survey). We tested if the estimated BC indices changed along the fishing gradient, i.e. if communities became increasingly different (or similar) from before to after fishing and across the fishing intensity gradient. Here we used Gaussian distribution GLMMs and tested three models, one including all BC indices as response variable and one for BC indices calculated from samples of each sediment type separately.

## Electronic supplementary material

Defining thresholds of sustainable impact on benthic communities in relation to fishing 1 disturbance
