# Supplementary material for: Defining thresholds of sustainable impact on benthic communities in relation to fishing disturbance
Source: Sci Rep. 2017 Jul 14;7:5440. doi: 10.1038/s41598-017-04715-4 (PMC5511154; doi:10.1038/s41598-017-04715-4)
Supplement: Supplementary file 1 — Defining thresholds of sustainable impact on benthic communities in relation to fishing 1 disturbance [file 41598_2017_4715_MOESM1_ESM.pdf]

Defining thresholds of sustainable impact on benthic communities in relation to fishing disturbance

Authors: GI Lambert, LG Murray, JG Hiddink, H Hinz, H Lincoln, N Hold, G Cambiè, MJ Kaiser

SUPPLEMENTARY INFORMATION S1

Table S1.1. List of infaunal taxa (family level) recorded during the 3 scientific surveys of the fishing impact experiment in Cardigan Bay (March, May and September 2014). Abundance is the total number of individuals recorded over the 235 grab samples taken. Occurrence is the percentage of grabs in which the taxa occurred. Taxa are organized by groups for which total biomass (in g) and occurrence are also given. Note that taxa were not weighed at the family level but at the group level only. Highlighted are the taxa present in ≥25% of samples taken.

Group	Taxa (Family level)	Abundance	Occurrence Taxa	Biomass Group	Occurrence Group
Bivalvia	Veneridae	165	0.31	2326.43	0.66
	Glycymerididae	116	0.23		
	Nuculidae	116	0.21		
	Mactridae	80	0.17		
	Cardiidae	38	0.09		
	Tellinidae	38	0.09		
	Semelidae	25	0.08		
	Anomiidae	19	0.01		
	Thraciidae	17	0.05		
	Pharidae	13	0.04		
	Hiatellidae	12	0.01		
	Astartidae	12	0.03		
	Pectinidae	11	0.02		
	Montacutidae	7	0.02		
	Psammobiidae	5	0.01		
	Ungulinidae	4	0.02		
	Kelliidae	3	0.01		
	Donacidae	2	0.01		
	Solenidae	2	0		
	Lucinidae	2	0		
	Arcticidae	1	0		
	Lasaeidae	1	0		
	Myidae	1	0		
	Mytilidae	1	0		
	Periplomatidae	1	0		

	Petricolinae	1	0		
Echinodermata	Ophiotrichidae<br>Ophiuridae<br>Amphiuridae<br>Cucumariidae<br>Synaptidae<br>Strongylocentrotidae<br>Fibulariidae<br>Asteriidae<br>Parechinidae	115<br>66<br>62<br>45<br>33<br>10<br>9<br>5<br>5	0.07<br>0.15<br>0.12<br>0.1<br>0.09<br>0.03<br>0.02<br>0.02<br>0.02	154.82	0.5
Polychaeta	Capitellidae<br>Lumbrineridae<br>Spionidae<br>Serpulidae<br>Terebellidae<br>Phyllodocidae<br>Glyceridae<br>Syllidae<br>Pectinariidae<br>Cirratulidae<br>Polynoidae<br>Nephtyidae<br>Pisionidae<br>Poecilochaetidae<br>Pholoidae<br>Maldanidae<br>Goniadidae<br>Sabellariidae<br>Nereididae<br>Sigalionidae<br>Dorvilleidae<br>Scalibregmatidae<br>Ampharetidae<br>Eunicidae<br>Opheliidae<br>Oweniidae<br>Sabellidae<br>Paraonidae<br>Flabelligeridae<br>Terebellidae<br>Chaetopteridae<br>Onuphidae	1512<br>630<br>523<br>379<br>369<br>331<br>266<br>236<br>206<br>178<br>106<br>106<br>92<br>75<br>72<br>68<br>65<br>58<br>50<br>49<br>48<br>46<br>41<br>40<br>38<br>34<br>30<br>29<br>29<br>25<br>16<br>15	0.67<br>0.62<br>0.49<br>0.17<br>0.43<br>0.32<br>0.48<br>0.34<br>0.12<br>0.32<br>0.23<br>0.31<br>0.1<br>0.15<br>0.15<br>0.17<br>0.15<br>0.07<br>0.13<br>0.17<br>0.11<br>0.17<br>0.06<br>0.11<br>0.1<br>0.09<br>0.09<br>0.08<br>0.03<br>0.06<br>0.05<br>0.03	108.66	0.98

	Orbiniidae	10	0.03		
	Polygordiidae	9	0.01		
	Trichobranchidae	9	0.03		
	Hesionidae	7	0.03		
	Magelonidae	7	0.02		
	Sphaerodoridae	4	0.02		
	Oenonidae	3	0.01		
Crustacea	Upogebiidae	243	0.28	107.01	0.7
	Cirolanidae	189	0.26		
	Mysidae	943	0.25		
	Dexaminidae	88	0.11		
	Ampeliscidae	60	0.17		
	Gnathiidae	58	0.09		
	Maeridae	38	0.07		
	Anthuridae	35	0.01		
	Photidae	34	0.06		
	Calliopiidae	29	0.05		
	Urothoidae	25	0.07		
	Paguridae	25	0.08		
	Atylidae	20	0.06		
	Copepoda *(Order)*	19	0.04		
	Diastylidae	17	0.06		
	Portunidae	17	0.07		
	Leucosiidae	17	0.05		
	Oedicerotidae	16	0.05		
	Aoridae	15	0.04		
	Porcellanidae	15	0.03		
	Corophiidae	15	0.04		
	Melitidae	13	0.03		
	Majidae	13	0.04		
	Verrucidae	9	0		
	Stenothoidae	9	0.03		
	Bodotriidae	8	0.03		
	Ischyroceridae	6	0		
	Balanidae	6	0.01		
	Crangonidae	5	0.01		
	Galatheidae	5	0.02		
	Thoracica (*Order*)	4	0.01		
	Unciolidae	4	0.01		
	Leucothoidae	4	0.02		
	Bathyporeiidae	3	0.01		
	Atelecyclidae	2	0.01		
	Brachyura	2	0.01		

	Callianassidae	2	0.01		
	Caprellidae	2	0.01		
	Isaeidae	2	0		
	Nebaliidae	2	0.01		
	Xanthidae	2	0.01		
	Amphilochidae	1	0		
	Thalassinidea	1	0		
Cnidaria	Cerianthidae	28	0.06	67.71	0.23
	Epizoanthidae	12	0.04		
	Sertulariidae	11	0.04		
	Campanulariidae	10	0.03		
	Edwardsiidae	5	0.02		
	Alcyoniidae	4	0.02		
	Tubulariidae	2	0.01		
	Plumulariidae	1	0		
Sipuncula	Golfingiidae	128	0.22	28.94	0.26
	Sipunculidae	11	0.02		
	Phascolosomatidae	1	0		
Bryozoa	Bitectiporidae	14	0.04	27.00	0.06
	Romancheinidae	5	0.01		
	Hippoporidridae	4	0.02		
	Crisiidae	3	0		
	Lichenoporidae	3	0		
	Flustridae	2	0.01		
	Alcyonidiidae	1	0		
	Escharinidae	1	0		
	Schizomavella	1	0		
	Electridae	1	0		
Porifera	Sycettidae	1	0	10.62	0.03
Nemertea	-	241	0.45	8.50	0.5
Chordata	Styelidae	9	0.02	7.79	0.11
	Molgulidae	9	0.04		
	Ammodytidae	2	0.01		
	Callionymidae	1	0		
	Didemnidae	1	0		
Gastropoda	Naticidae	11	0.03	6.40	0.08
	Eulimidae	3	0.01		
	Buccinidae	3	0.01		
	Tritoniidae	3	0		
	Clionidae	2	0.01		
	Proctonotidae	1	0		
	Pyramidellidae	1	0		
	Scaphandridae	1	0		

	Trochidae<br>Muricidae	1<br>1	0<br>0		
Nematoda	-	91	0.14	0.04	0.14
Polyplacophora	Leptochitonidae	36	0.08	0.54	0.09
Cephalochordata	Branchiostomidae	18	0.06	2.78	0.04
Phoronida	Phoronida	15	0.03	0.09	0.03
Platyhelminthes	Turbellaria *(Class)*	6	0.01	0.09	0.03
Annelida	Enchytraeidae	11	0.02	0.08	0.02
Mollusca	Neomeniidae	1	0	0.02	0
Chelicerata	Ammotheidae	1	0	0.00	0

Table S1.2. List of epifaunal taxa (species and gender level) recorded during the 3 scientific surveys of the fishing impact experiment in Cardigan Bay (March, May and September 2014). Abundance is the total number of individuals recorded over the 206 beam trawl samples taken. Biomass is in kilograms. Occurrence is the percentage of tows in which the taxa occured. Numbers are given at the taxa and group level. Highlighted are the taxa present in ≥25% of samples taken.

		Species			Group		
Group	Species	Abundance	Biomass	Occurrence	Abundance	Biomass	Occurrence
Echinodermata	*Ophiothrix fragilis*	230274	303.969	0.9	243603	369.267	0.99
	Psammechinus miliaris	4626	6.9884	0.92			
	Ophiura albida	4144	1.6853	0.82			
	Asterias rubens	4020	55.115	0.88			
	Ophiura ophiura	442	1.098	0.51			
	Ophiocomina nigra	83	0.2835	0.08			
	Holothuria spp	6	0.058	0.03			
	Anseropoda placenta	3	0.01	0.01			
	Henricia oculata	2	0.012	0.01			
	Astropecten irregularis	1	0.015	0			
	Crossaster papposus	1	0.001	0			
	Echinus esculentus	1	0.032	0			
Crustacea	*Pagurus bernhardus*	2056	25.8344	0.95	10462	119.417	0.99
	Pagurus prideauxi	1964	9.5856	0.88			
	Inachus spp	919	1.676	0.67			
	Macropodia spp	841	0.6481	0.67			
	Crangon crangon	703	0.7474	0.56			
	Ebalia spp	585	0.5143	0.68			
	Liocarcinus holsatus	422	1.4975	0.5			
	Palaemon spp	369	0.6028	0.26			
	Xantho pilipes	359	0.6333	0.57			
	Inachus dorsettensis	356	0.859	0.19			
	Liocarcinus depurator	322	1.9853	0.49			
	Hyas spp	279	0.3835	0.44			
	Pandalus spp	232	0.269	0.31			
	Eurynome spp	227	0.4653	0.42			
	Pisidia longicornis	174	0.106	0.37			
	Necora puber	170	5.2848	0.35			
	Liocarcinus pusillus	146	0.14	0.32			
	Maja squinado	89	54.843	0.28			
	Galathea spp	60	0.434	0.15			
	Atelecyclus rotundatus	42	0.412	0.15			

	Cancer pagurus	39	12.127	0.17			
	Munida rugosa	16	0.203	0.06			
	Rissoides desmaresti	11	0.0413	0.04			
	Upogebia sp	11	0.024	0.05			
	Cirolana cranchi	6	0.013	0.02			
	Anapagurus laevis	5	0.007	0.02			
	Conilera cylindracea	4	0.003	0.01			
	Hippolytidae sp	4	0.002	0.01			
	Liocarcinus arcuartus	4	0.005	0.01			
	Gammarus sp	2	0.002	0.01			
	Pilumnus hirtellus	2	0.003	0			
	Dromia personata	1	0.032	0			
	Pontophilus spinosus	1	0.001	0			
Bivalvia	*Pecten maximus*	5437	1111.627	0.83	6548	1137.201	0.94
	Glycymeris glycymeris	317	12.8959	0.3			
	Aequipecten opercularis	247	5.3984	0.34			
	Clausinella fasciata	175	0.4877	0.27			
	Tapes rhomboides	130	2.1613	0.26			
	Laevicardium crassum	74	2.4484	0.21			
	Palliolum tigerinum	29	0.643	0.09			
	Spisula elliptica	26	0.041	0.07			
	Timoclea ovata	20	0.024	0.07			
	Arcopagia crassa	20	0.891	0.06			
	Astarte sulcata	16	0.085	0.06			
	Nucula nucleus	11	0.011	0.04			
	Chlamys varia	10	0.042	0.02			
	Dosinia exoleta	9	0.1132	0.03			
	Gari spp	6	0.014	0.01			
	Circomphalus casina	6	0.176	0.02			
	Anomia ephippium	6	0.016	0.03			
	Chlamys distorta	2	0.009	0.01			
	Arctica islandica	2	0.075	0.01			
	Modiolarca tumida	1	0.006	0			
	Acanthocardia tuberculata	1	0.032	0			
Cnidaria	*Alcyonium digitatum*	3213	47.4036	0.89	5245	58.486	1
	Adamsia carciniopados	1371	6.6208	0.68			
	Nemertesia spp	-	0.595	0.46			
	Metridium senile	134	3.307	0.33			
	Hydrallmania spp	-	0.1281	0.44			
	Abietinaria abietina	-	0.109	0.35			
	Sertularella gayi	-	0.05	0.13			
	Sertularia sp	-	0.048	0.11			
	Urticina felina	10	0.161	0.03			
	Tubularia indivisa	-	0.026	0.04			
	Halecium halecium	-	0.002	0.01			

	Sagartia sp	-	0.006	0.01			
Chordata	*Callionymus lyra*	1683	39.2827	0.92	4385	123.755	0.96
	Trisopterus minutus	655	6.7217	0.76			
	Microchirus variegatus	406	7.9476	0.68			
	Phrynorhombus norvegicus	276	2.1708	0.54			
	Merlangius merlangus	263	14.0196	0.39			
	Ammodytes spp	98	1.25	0.24			
	Arnoglossus laterna	97	1.343	0.27			
	Aspitrigla cuculus	90	5.1047	0.28			
	Blennius ocellaris	89	1.728	0.28			
	Ctenolabrus rupestris	89	1.921	0.23			
	Agonus cataphractus	87	0.718	0.29			
	Limanda limanda	68	3.9014	0.22			
	Gaidropsarus vulgaris	65	0.5175	0.16			
	Eutriglia gurnardus	47	0.883	0.13			
	Pomatoschistus minutus	44	0.033	0.09			
	Gobidae spp	41	0.021	0.04			
	Scyliorhinus canicula	36	13.948	0.13			
	Diplecogaster bimaculata	34	0.121	0.11			
	Microstomus kitt	29	1.0539	0.11			
	Pholis gunnellus	27	0.18	0.08			
	Gadus morhua	25	2.9229	0.1			
	Trigla lucerna	18	0.758	0.06			
	Myoxocephalus scorpius	16	1.019	0.06			
	Pleuronectes platessa	14	1.442	0.06			
	Raja montagui	10	4.854	0.05			
	Echiichthys vipera	9	0.158	0.02			
	Trisopterus luscus	9	0.566	0.02			
	Lepadogaster candollei	8	0.008	0.03			
	Raja brachyura	7	1.884	0.03			
	Scyliorhinus stellaris	7	4.528	0.02			
	Liparis montagui	5	0.048	0.02			
	Syngnathus acus	5	0.037	0.02			
	Trachinus draco	5	0.207	0.01			
	Lophius piscatorius	4	1.395	0.02			
	Pomatoschistus pictus	4	0.006	0.02			
	Chirolophis ascanii	3	0.095	0.01			
	Raja clavata	3	0.383	0.01			
	Solea solea	2	0.198	0.01			
	Buglossidium luteum	1	0.001	0			
	Conger sp	1	0.04	0			
	Lesueurigobius friesii	1	0.001	0			
	Mullus surmuletus	1	0.009	0			
	Mustelus asterias	1	0.147	0			
	Taurulus bubalis	1	0.064	0			

	Trisopterus esmarkii	1	0.118	0			
Gastropoda	*Aporrhais pespelecani*	655	4.909	0.39	1736	42.915	0.87
	Buccinum undatum	412	33.7782	0.58			
	Calliostoma zizyphinum	210	0.6146	0.44			
	Ocenebra erinacea	184	0.7243	0.44			
	Colus gracilis	64	2.013	0.21			
	Trivia sp	64	0.048	0.16			
	Hinia reticulata	25	0.024	0.08			
	Polinices spp	25	0.045	0.08			
	Archidoris pseudoargus	22	0.11	0.1			
	Tritonia hombergi	20	0.074	0.07			
	Mangelia attenuata	14	0.015	0.05			
	Capulus ungaricus	9	0.025	0.04			
	Epitonium clathrus	7	0.01	0.03			
	Diodora graeca	4	0.006	0.02			
	Doto fragilis	3	0.125	0.01			
	Scaphander lignarius	3	0.032	0.01			
	Emarginula fissura	2	0.003	0.01			
	Gibbula sp	2	0.002	0.01			
	Neptunea antiqua	2	0.346	0.01			
	Aeolidia papillosa	1	0.001	0			
	Crepidula fornicata	1	0.002	0			
	Melanella spp	1	0.001	0			
	Onchidaris bilamellata	1	0.001	0			
Bryozoa	*Pentapora foliacea*	-	1.308	0.15	380	4.728	0.58
	Flustra foliacea	-	2.8111	0.45			
	Cellaria sp	-	0.3972	0.24			
	Alcyonidium diaphanum	-	0.175	0.09			
	Bugula sp	-	0.002	0.01			
Polychaeta	*Aphrodita aculeata*	66	0.514	0.21	107	0.566	0.33
	Nepthys spp	19	0.0152	0.05			
	Polychaete spp	18	0.031	0.08			
	Lanice conchilega	3	0.004	0.01			
	Sabella pavonina	1	0.002	0			
Porifera	*Dysidea fragilis*	39	0.586	0.14	103	0.936	0.24
	Raspalia ramosa	30	0.152	0.07			
	Suberites domuncula	15	0.088	0.04			
	Tethya citrina	9	0.032	0.01			
	Axinella dissimilis	3	0.016	0.01			
	Axinella sp	3	0.006	0.01			
	Porifera spp	3	0.028	0.01			
	Suberites carnosus	1	0.028	0			
Ascidian	*Ciona intestinalis*	38	0.473	0.11	62	0.772	0.18
	Botryllus sp	1	0.031	0			
Cephalopoda	*Eledone cirrhosa*	19	1.35	0.09	28	1.375	0.12

	Sepiola atlantica	7	0.023	0.03			
Polyplacophora	*Leptochiton asellus*	17	0.01	0.05	17	0.010	0.05
Scaphopoda	*Antalis entalis*	17	0.0152	0.05	17	0.015	0.05
Nemertea	*Tubulanus annulatus*	1	0.001	0	1	0.001	0
Sipuncula	*Golfingia vulgaris*	1	0.001	0	1	0.001	0

SUPPLEMENTARY MATERIAL S2

Table S2.1. Description of the statistical distributions used to test the effect of fishing intensity on epifauna and infauna

**Sampling**	**Level**	**Variable**	**Distribution**	**Number of groups analysed**	**Note**
Infauna	Total	Abundance	Lognormal	1	
Infauna	Total	Biomass	Lognormal	1	
Epifauna	Total	Abundance	Lognormal	1	
Epifauna	Total	Biomass	Lognormal	1	
Infauna	Class/ Phylum	Biomass	Lognormal	6	consitutes 96% of total biomass
Epifauna	Class/ Phylum	Biomass	Lognormal	7	consitutes 99% of total biomass[excluding *P. maximus* and *O. fragilis]*
Infauna	Class/ Phylum	Abundance	Poisson (or NB)		
Epifauna	Class/ Phylum	Abundance	Poisson (or NB)		
Infauna	Family	Abundance	Poisson (or NB)	1	only one abundant family (Capitellidae)
Infauna	Family	Abundance	Binomial(log-log)	12	for all other species in > 25% of samples (leaving out zero-inflated datasets) (Zuur et al., 2009).
Epifauna	Species/Gender	Abundance	Poisson (or NB)	16	defined as taxa observed during each survey and for which the average positive density over all sites was >4 individuals $100m^{-2}$, cut-off defined from preliminary data exploration
Epifauna	Species/Gender	Abundance	Binomial (log-log)	28	for all other species in > 25% of samples (leaving out zero-inflated datasets) (Zuur et al., 2009).
Epifauna	Species/Gender	Biomass	Gamma (log)	16	only for most abundant species as other species analysed as presence-absence, Gamma was preferred over log-normal only for logistical reasons, to use the glmer(lme4) function that was used for other species

Table S2.2. Description of a number of published experimental studies presenting their effect size and statistical power, compared to the present study. Total impact area is estimated from the dimensions and fishing intensity of the experiments, i.e. Dimension x Intensity (= area x number of times area entirely fished) = Total impact area. α is the type-I error rate assumed by the study. Effect size is either the effect size tested or the detectable effect size for a power of 80%(*) (adapted from Leblanc et al. 2015).

Study	Dimension	Impact gear	Intensity	Total impact area (km^2)	α	Effect size (%)	Power (%)
Currie and Parry (1996)	one 0.6 x 0.6 km plot	3m wide Peninsula dredges	2	0.7	0.1	30	11–47
Currie and Parry (1999); site 1	one 0.6 x 0.6 km plot	3m wide Peninsula dredges	2	0.7	0.05	30	12–47
Currie and Parry (1999); site 2	one 0.6 x 0.6 km plot		4	1.4	0.05	30	17–72
Pitcher et al. (2009); epibenthic dredge sampling	12 pairs, 2.8 x 1.2km	18.5 and 37m prawn trawl	1	20.2	0.05	45–89	80(*)
Pitcher et al. (2009); prawn trawl sampling			1		0.05	34–89	80(*)
Ragnarsson and Lindegarth (2009)	4 pairs, 1 x 0.1km	otter-trawl, swath range 80-120m	10	2.0	0.05	25	10–100
McConnaughey and Syrjala (2014) - immediate effect	6 pairs of corridors, 20.9 x 0.1km	Aleutian combination otter trawl	4	50.2	0.1	26 - 462	80(*)
McConnaughey and Syrjala (2014) - after 1 year			4		0.1	35 - 448	80(*)
Leblanc et al (2015); site 1 (grab sampling)	15 plots, 0.4 x 0.04km (+ one control)	4.0 m wide Digby-type rock dredge	1–7	1.7	0.05	22	22 - 54
Leblanc et al (2015); site 1 (video)			0–14		0.05	22	38 - 79
Leblanc et al (2015); site 2 (grab sampling)	15 plots, 0.4 x 0.04km (+ one control)		0–12	1.4	0.05	22	9 - 93
Leblanc et al (2015); site 2 (video)			0–12		0.05	22	100
This study; grab abundance - class/phylum level (presence at family level)	10 plots (+ 3 control), 1.7 x 0.37km	0.76m Newhaven dredge	0 - 6	19.7	0.1	25	32 – 95 (13-82)
This study; grab biomass - class/phylum level			0 - 6		0.1	25	25 - 78
This study; beam trawl abundance - class/phylum level (species level)	13 plots (+ 4 controls), 1.7 x 0.37km		0 - 6		0.1	25	60 – 100 (31 – 99)
This study; beam trawl biomass - class/phylum level (species level)			0 - 6		0.1	25	61 – 100 (20 – 97)

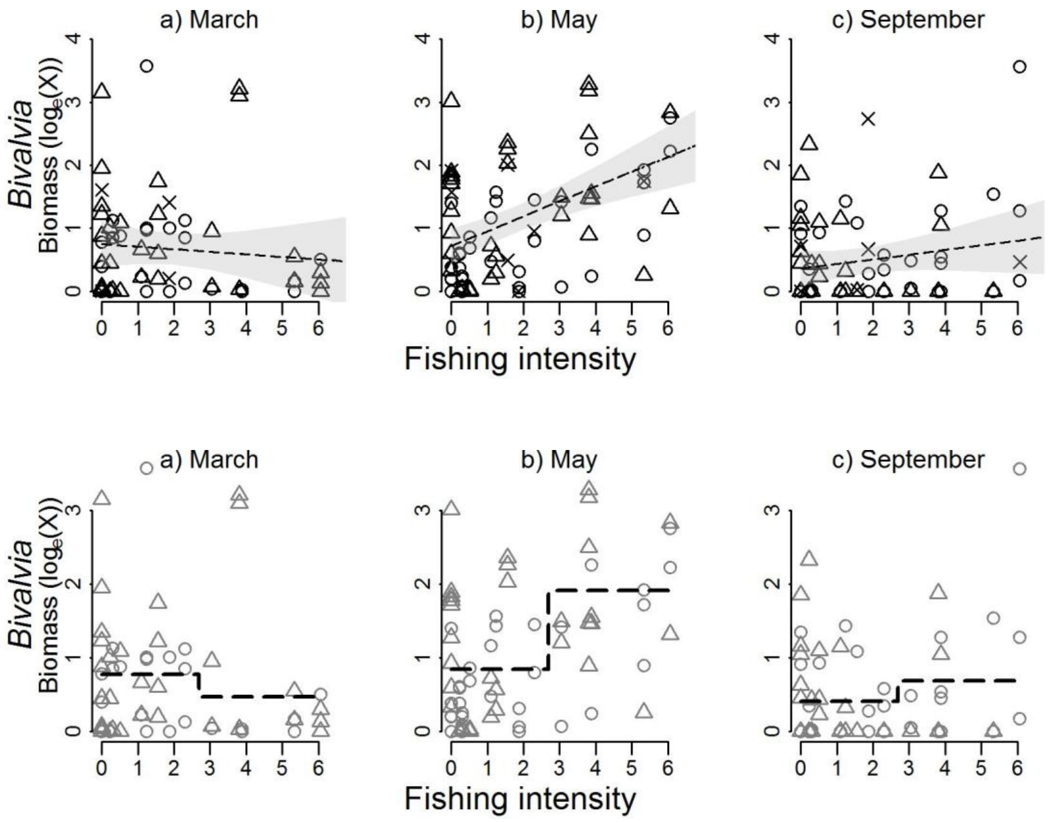

Figure S2.1. Effect of scallop dredging on epifaunal biomass by group (excluding *Pecten maximus* and *Ophiothrix fragilis*). Left to right: March (prior to dredging), May (after dredging) and September (4 months after dredging). X is measured in kgs per 100m^2 x100+1. Each symbol is a sample, distinguishing sand (o) and gravel (△) sediment types, and sites where no sediment information was available (x) are shown on the first plots (data not included in the models). Two model outputs are presented for each group of species, first the GLMM fits with continuous fishing intensity as predictor, second the GLMM fits with categorical fishing intensity (low vs high) as predictor. Lines show the predicted values from best models (dash line for sand and dotted line for gravel) with 95% confidence intervals for the GLMM with continuous fishing intensity (grey shading). Confidence intervals are not shown on the categorical fishing GLMMs for ease of visual interpretation.

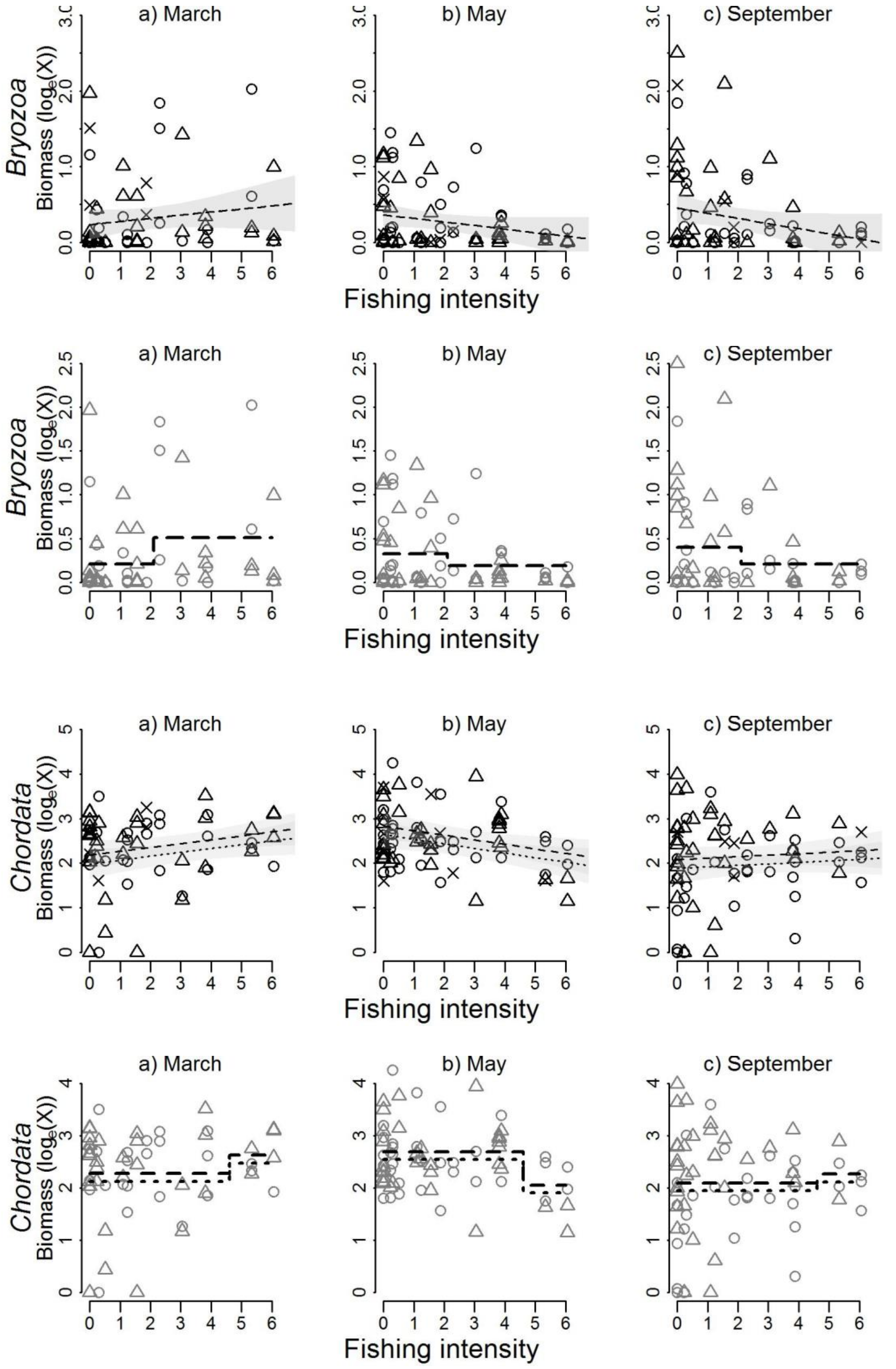

Figure S2.1. (continued)

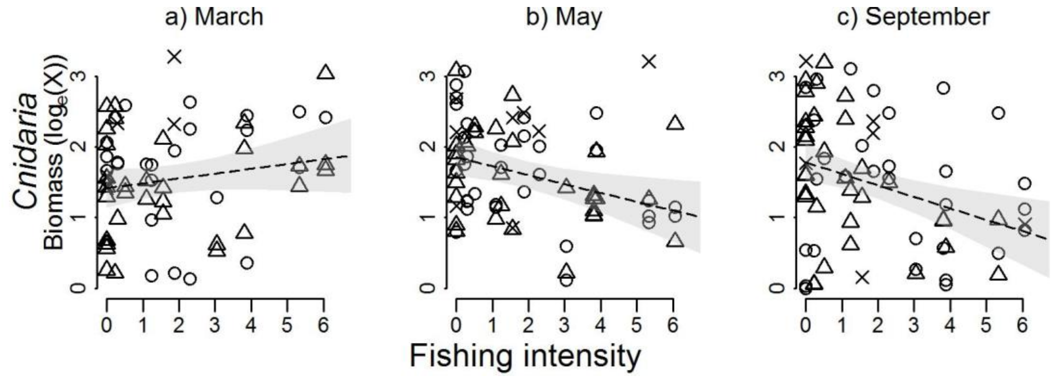

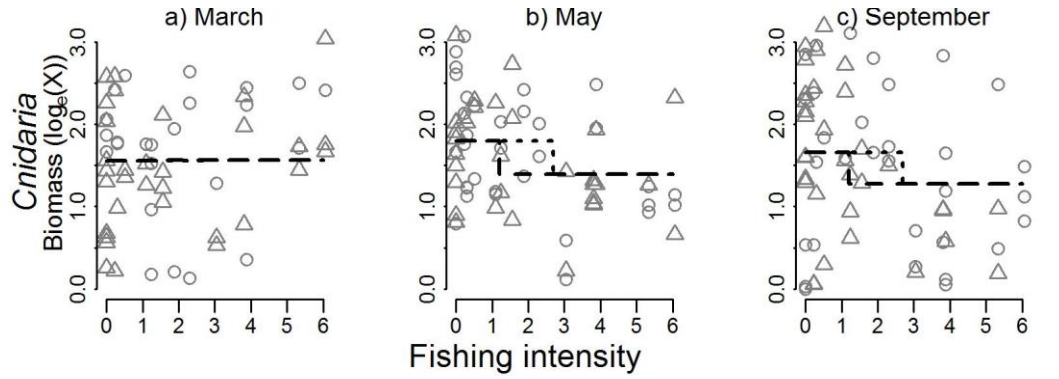

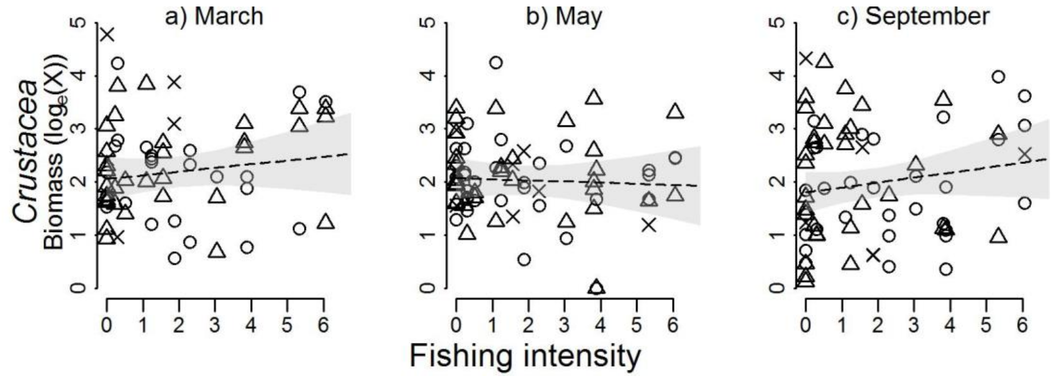

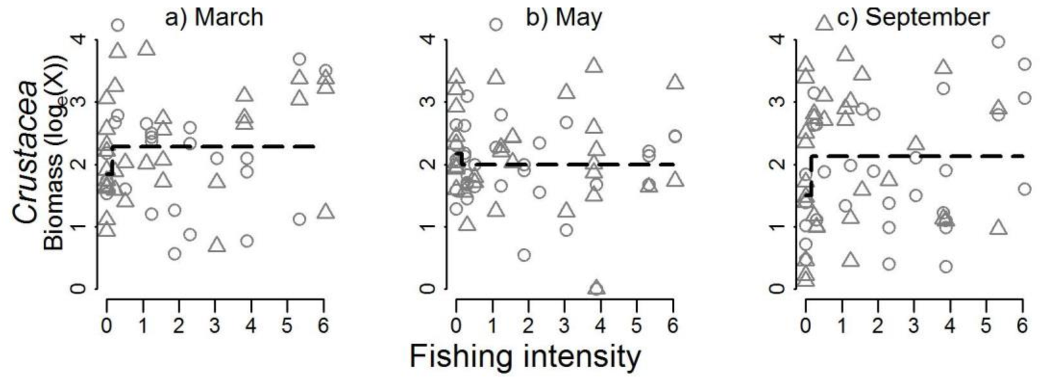

Figure S2.1. (continued)

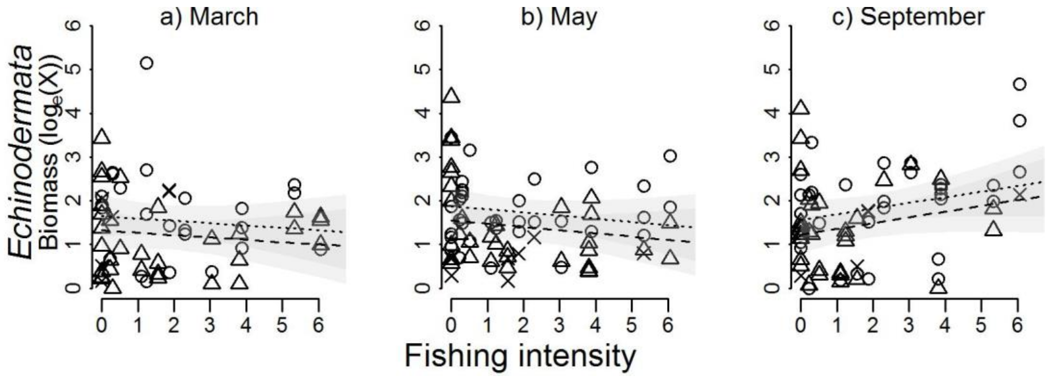

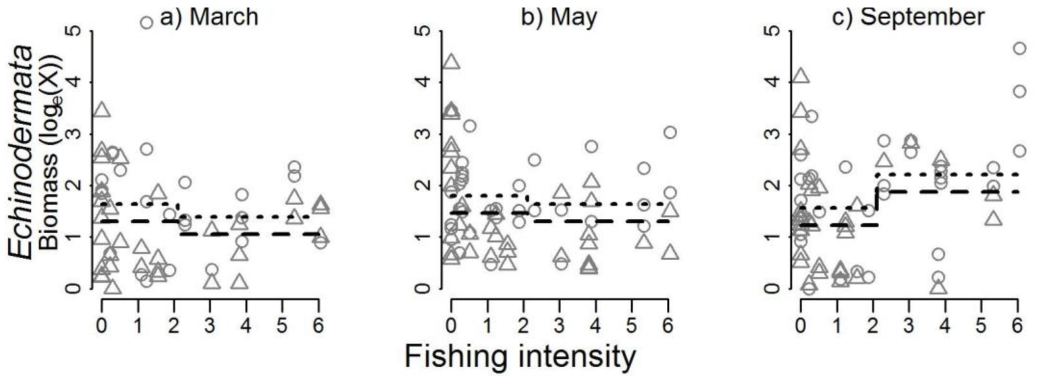

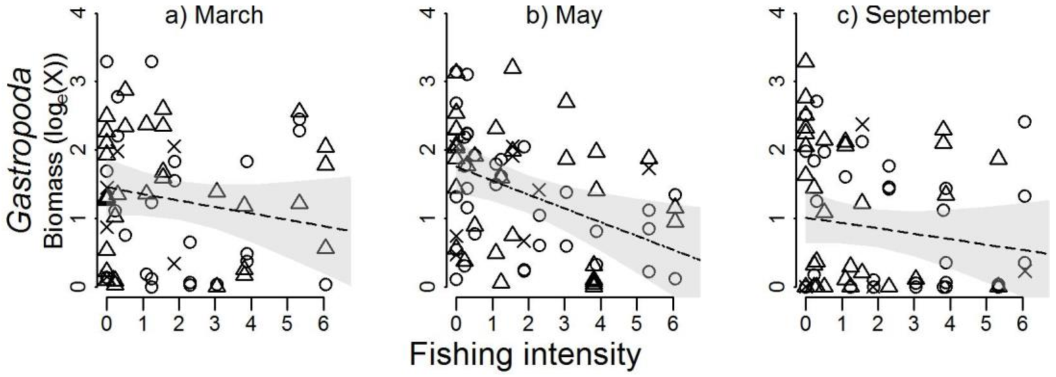

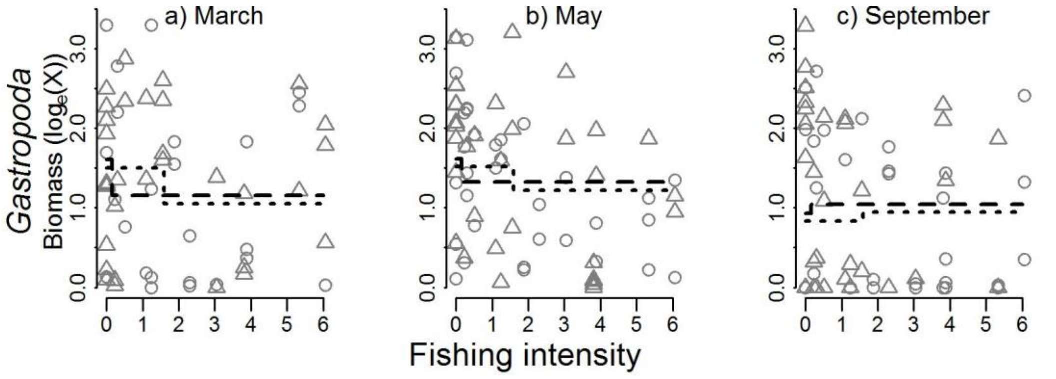

Figure S2.1. (end)

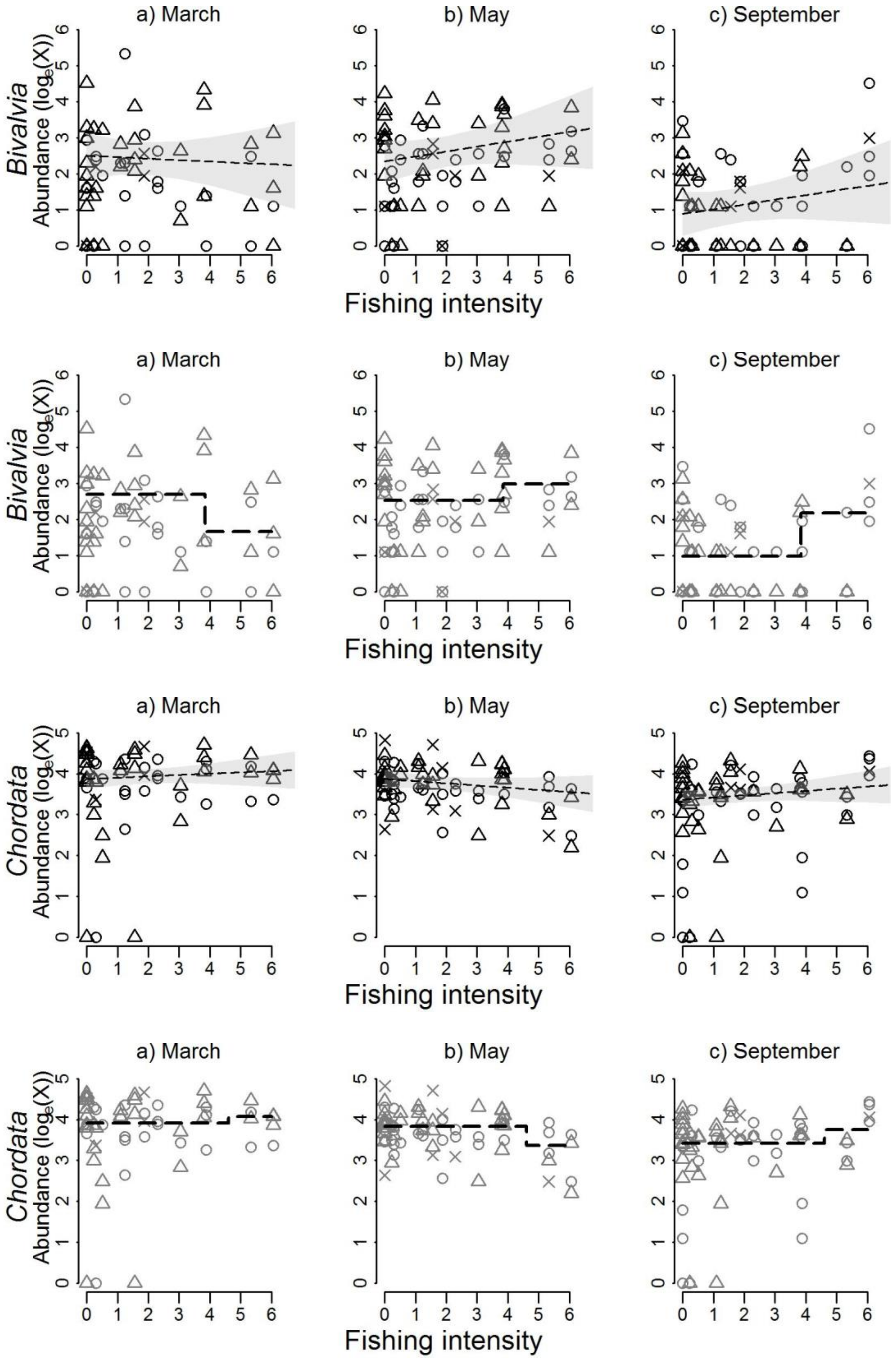

Figure S2.2. Effect of scallop dredging on epifaunal abundance by group (excluding *P. maximus* and *O. fragilis* from the epifaunal analysis). X is measured in numbers per 100m^2 x10+1. See also legend Figure S2.1.

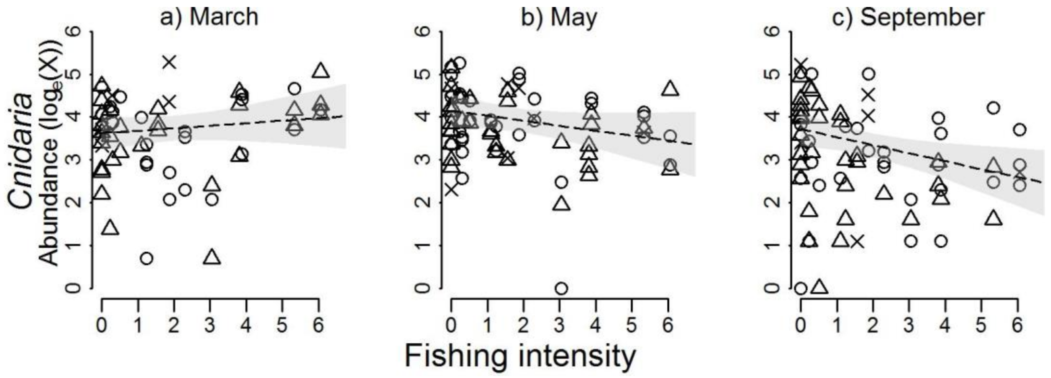

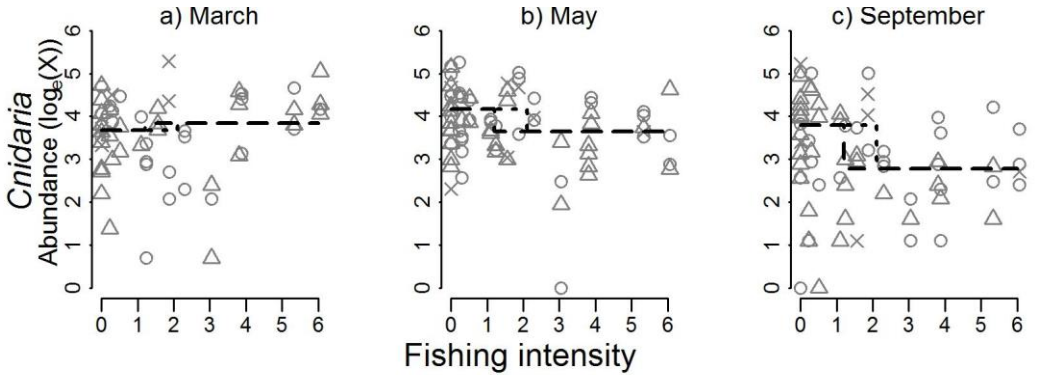

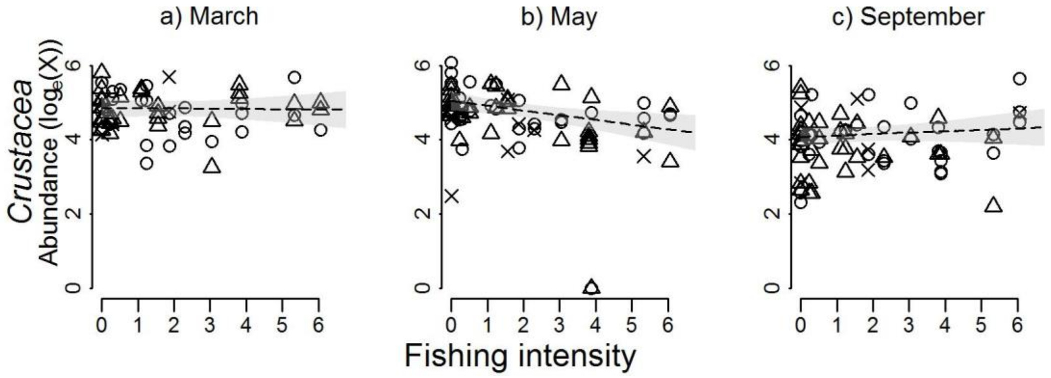

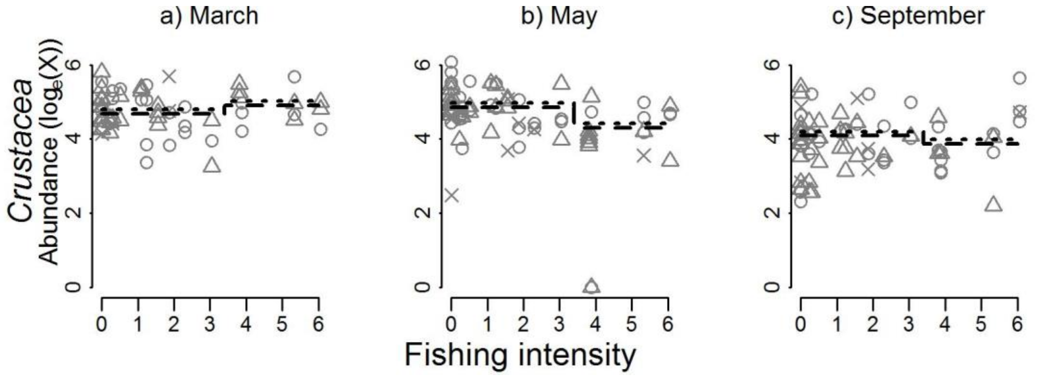

Figure S2.2. (continued).

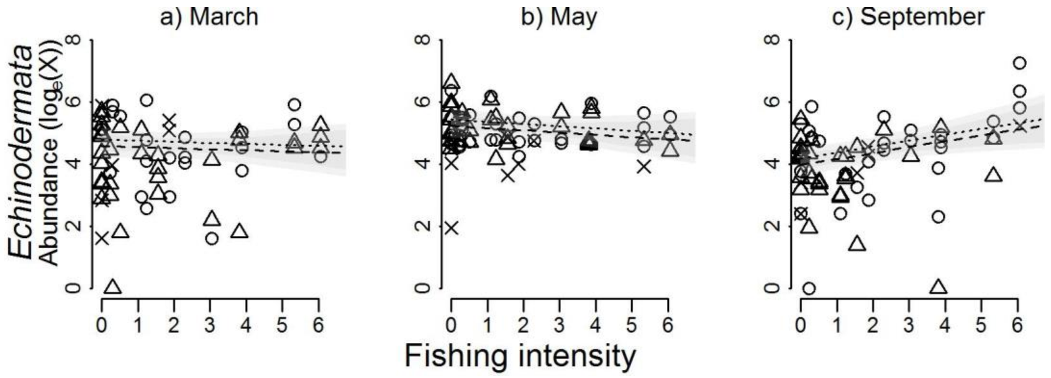

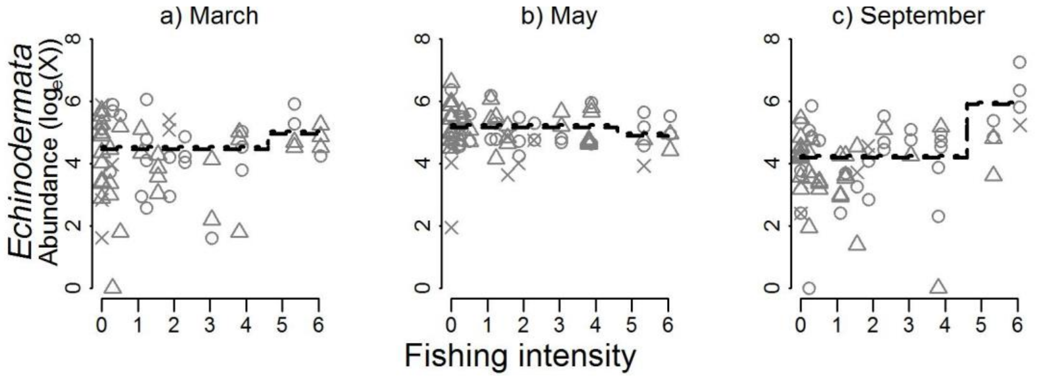

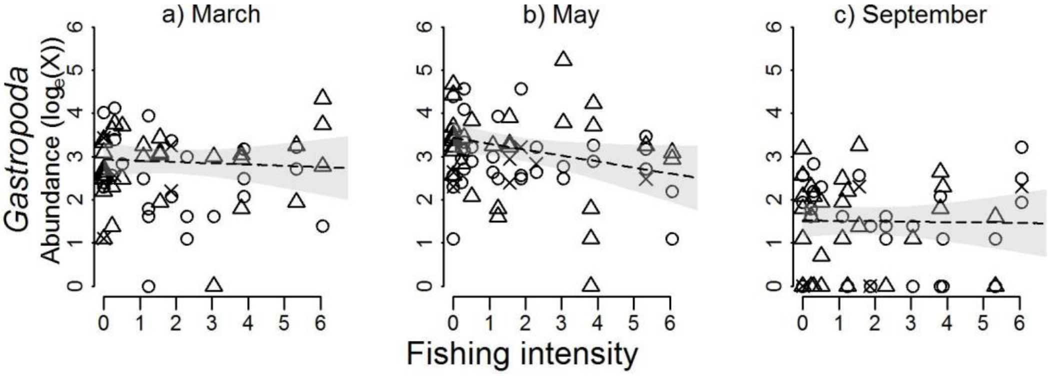

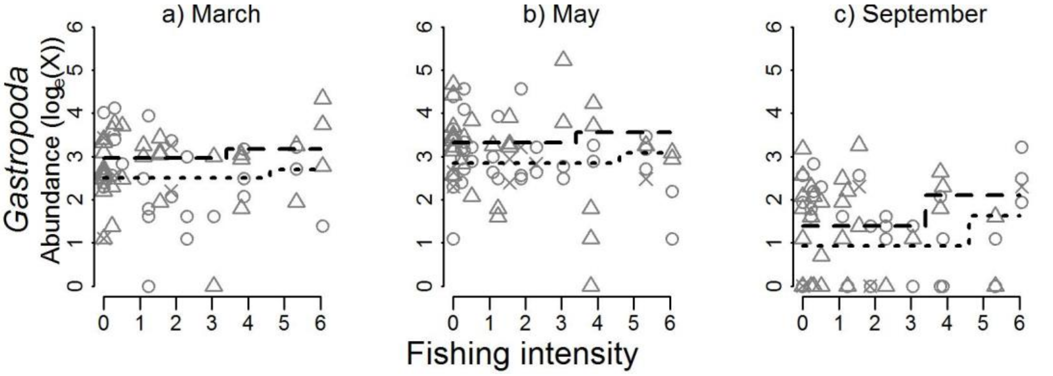

Figure S2.2. (end).

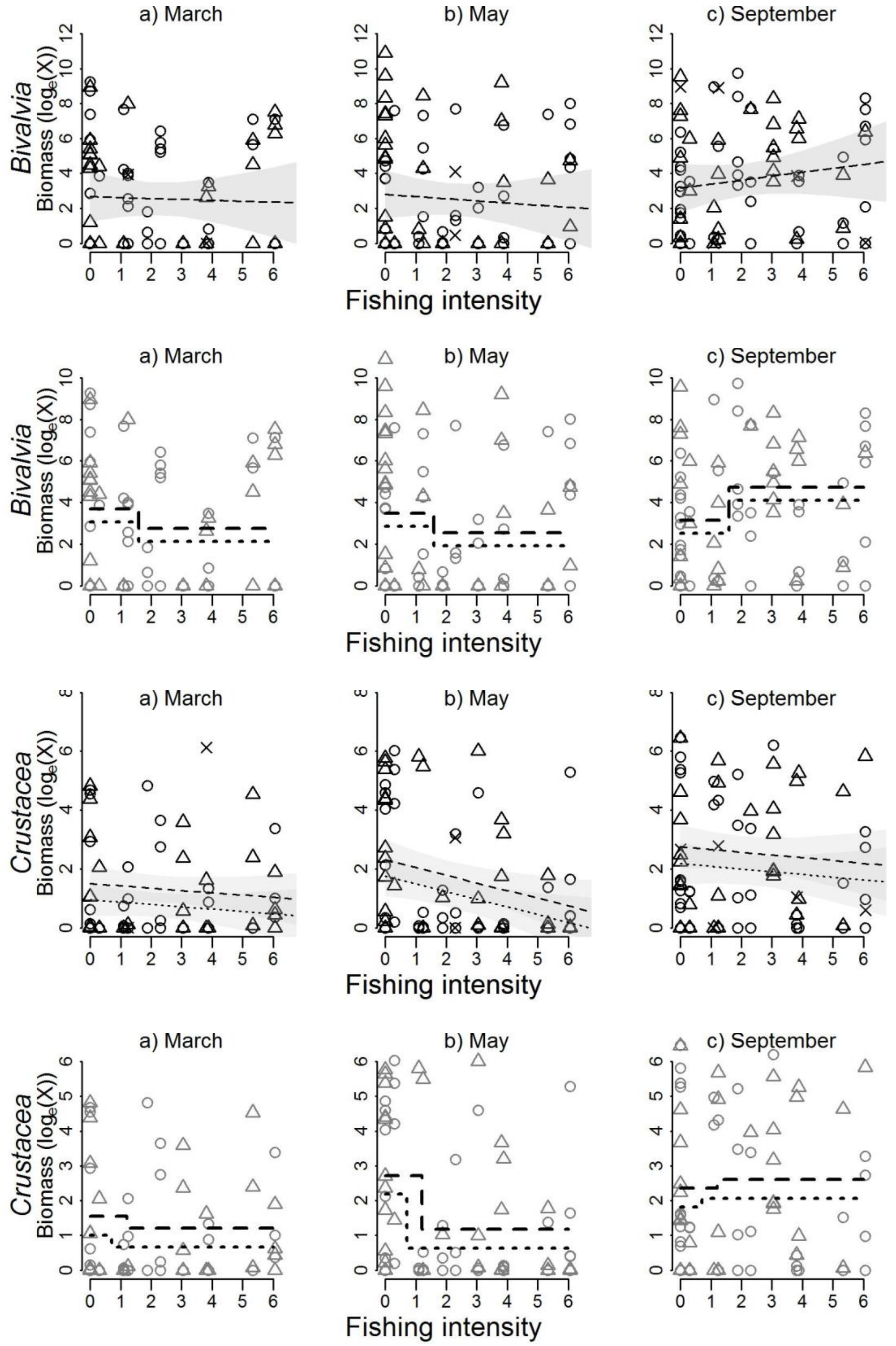

Figure S2.3. Effect of scallop dredging on infaunal biomass by group. X is measured in g per 0.1m^2 x100+1. See also legend Figure S2.1.

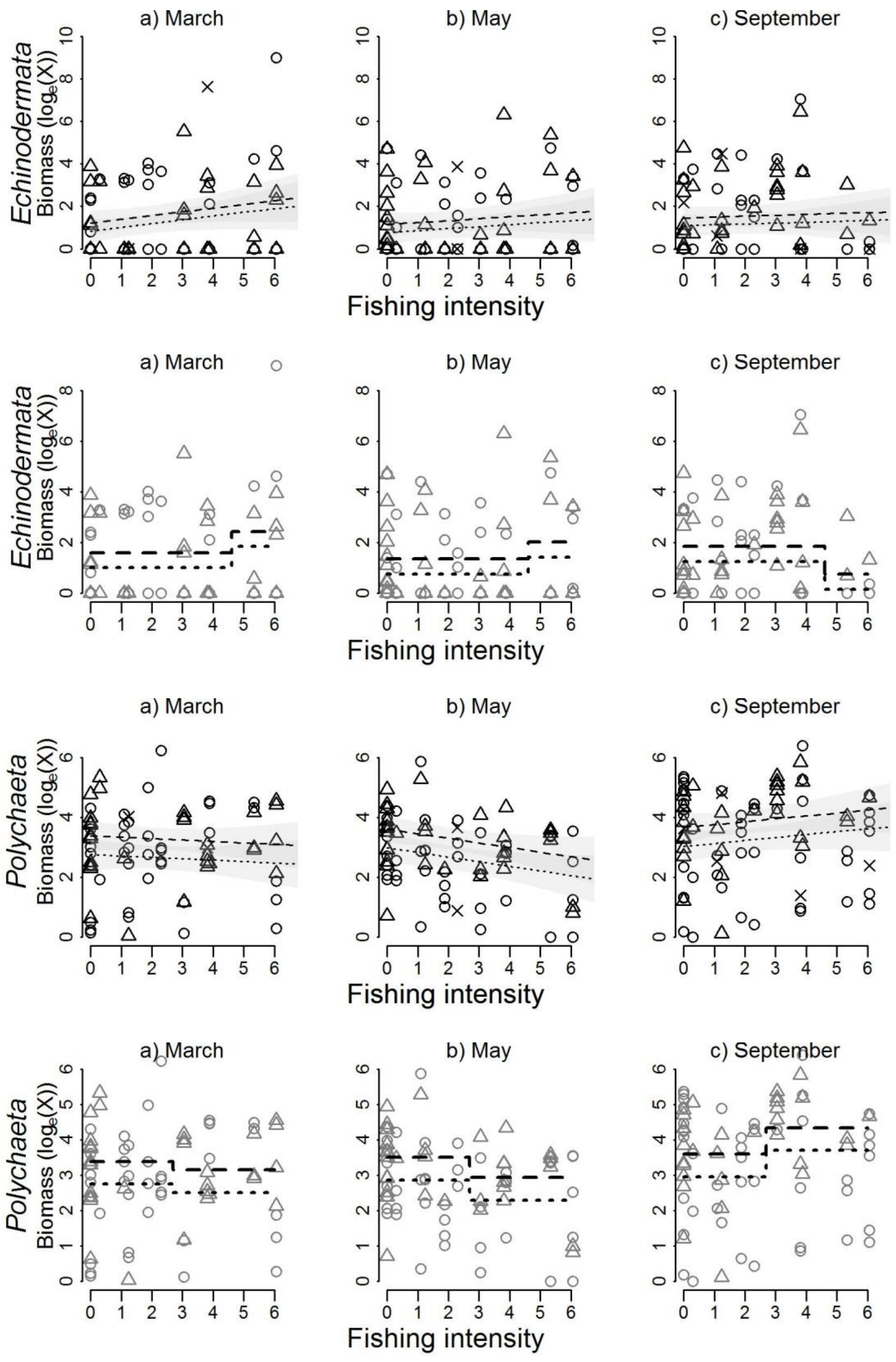

Figure S2.3 (continued).

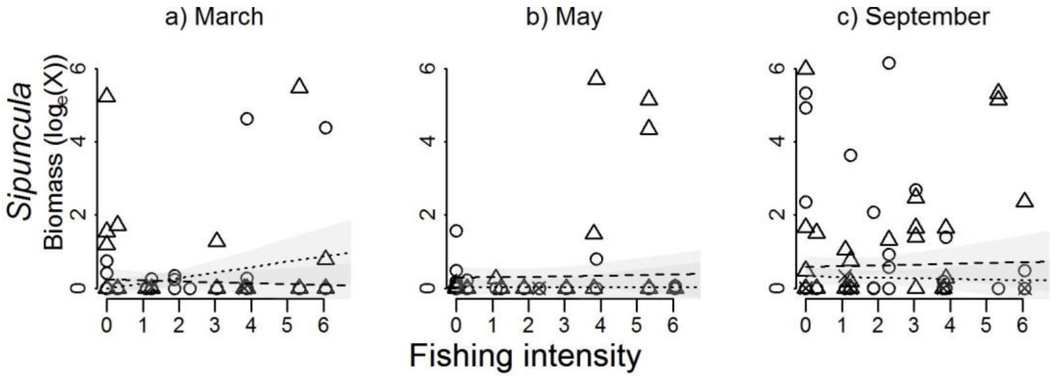

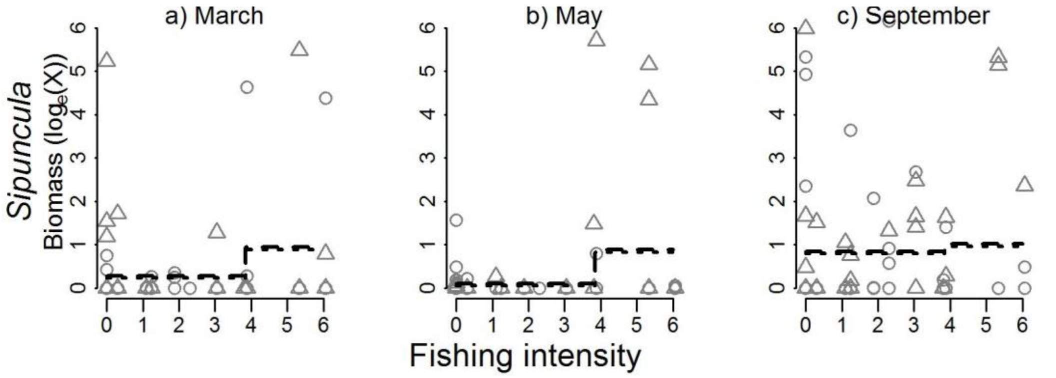

Figure S2.3 (end).

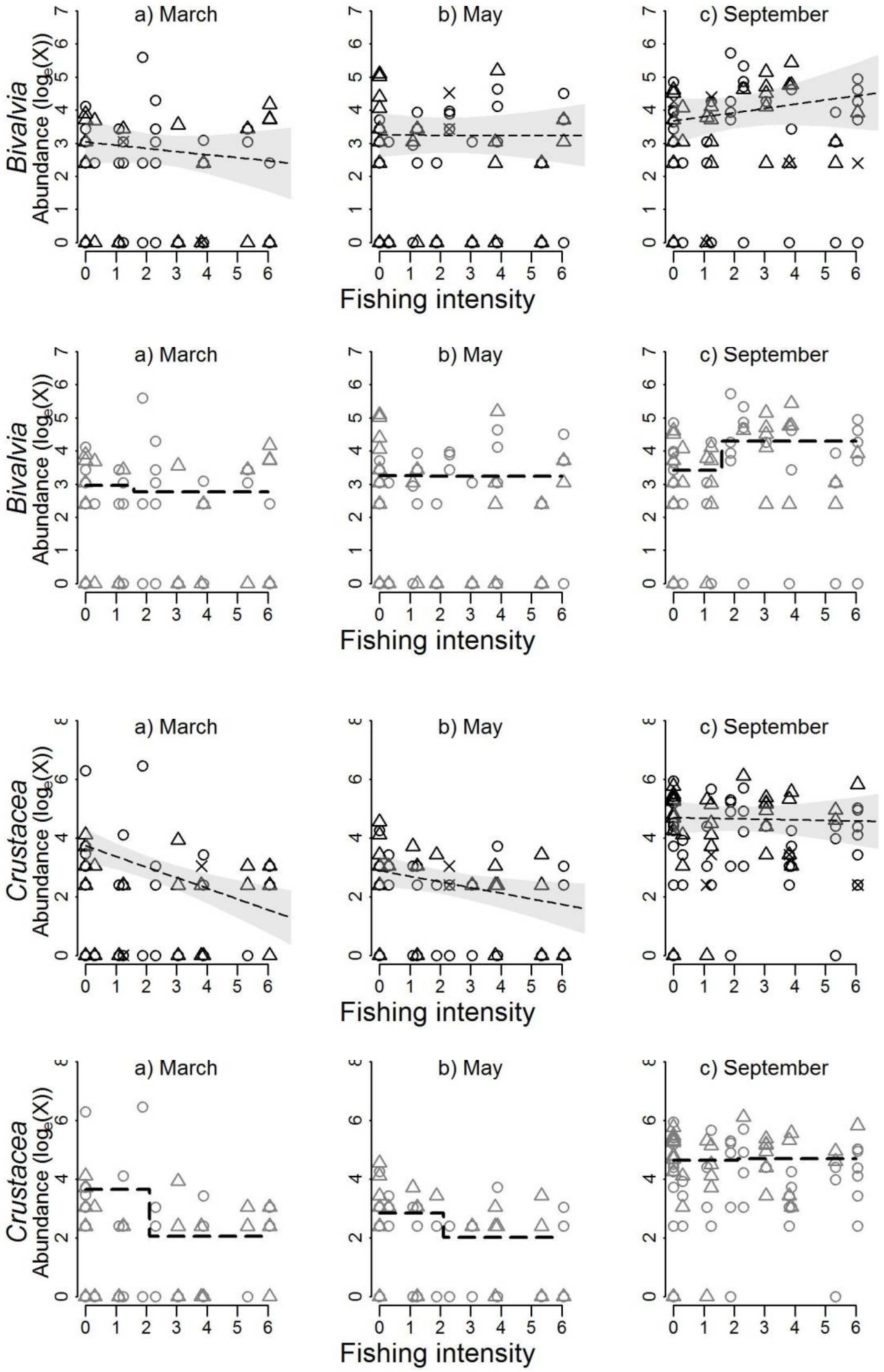

Figure S2.4. Effect of scallop dredging on infaunal abundance by group. X is measured in number per 0.1m^2 x10+1. See also legend Figure S2.1.

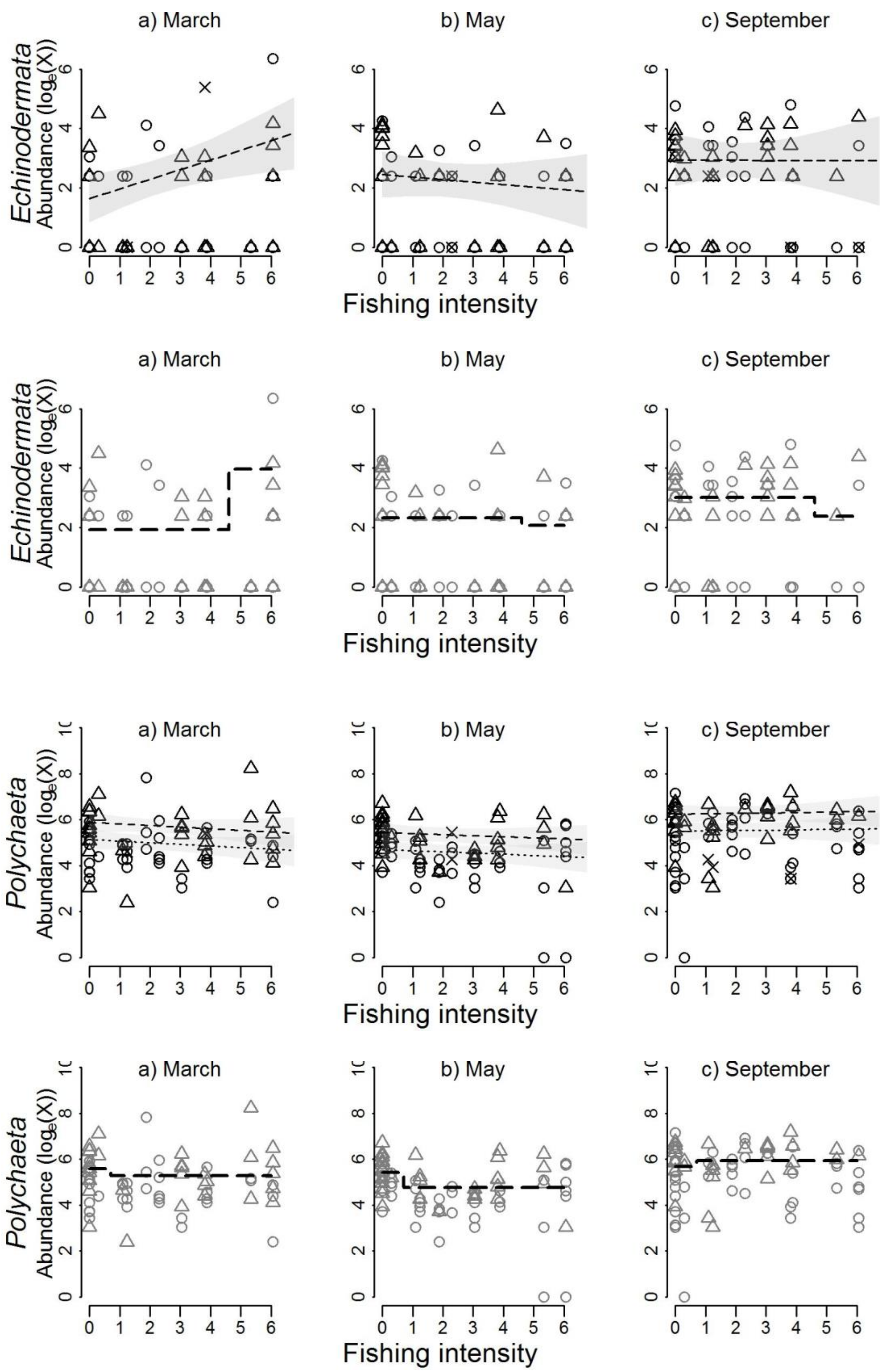

Figure S2.4 (continued).

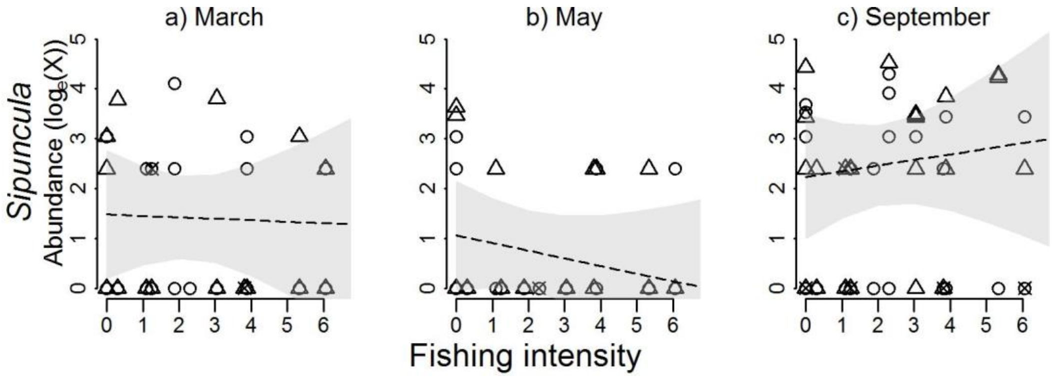

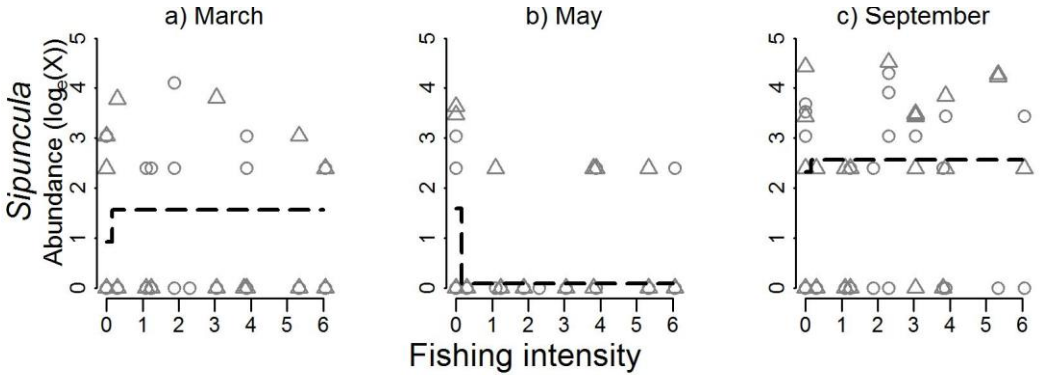

Figure S2.4 (end).

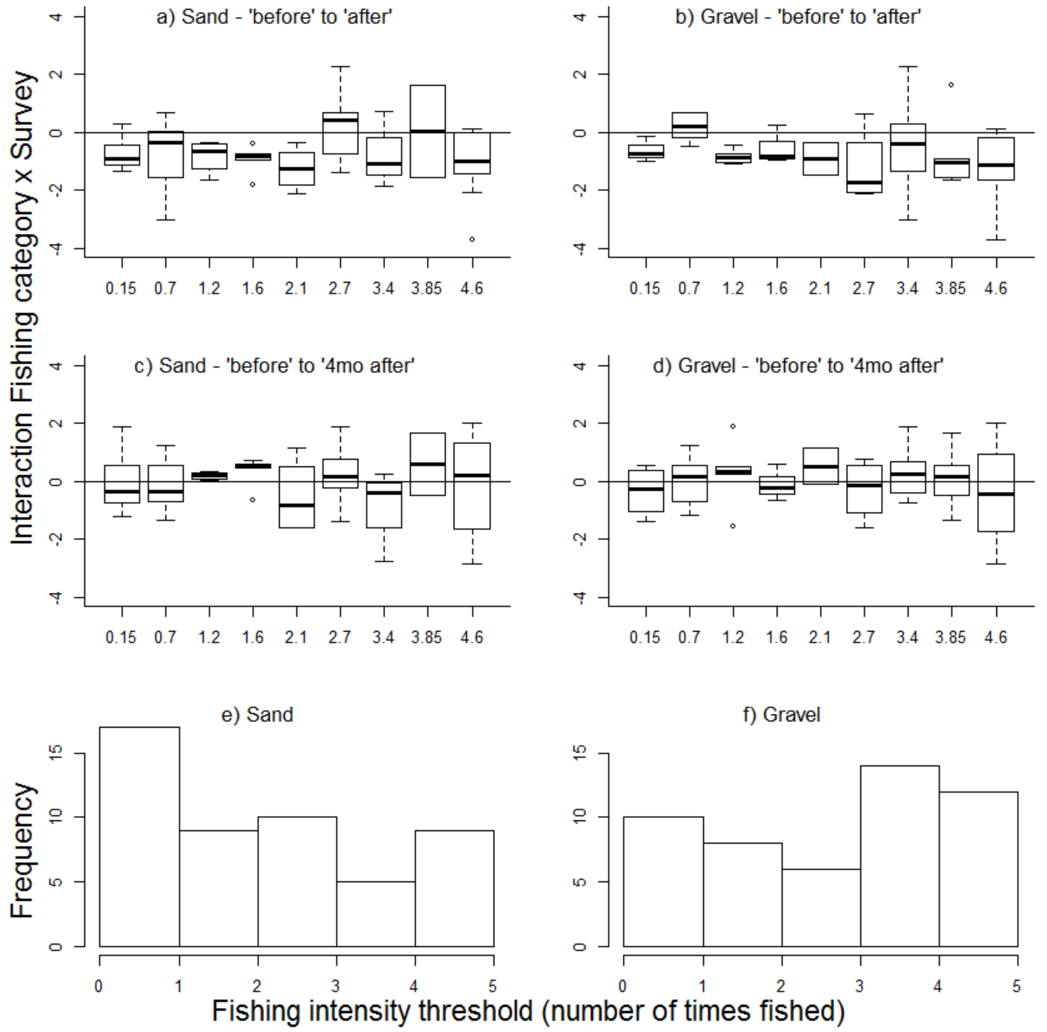

Figure S2.5. Effect of taxa-specific fishing intensity (FI) thresholds on epifaunal and infaunal abundance and presence (on log-transformed data, using odds for presence). The term effect here corresponds to the estimate reported from the models (i.e. log-scale percentage change from before to after fishing). A single FI threshold was identified for each species combining all three surveys, and for some species the FI threshold was specific to sediment type. When it was not, the effect and threshold were displayed in both sand and gravel panels here (a-d). The y-axis shows the estimated interaction between FI category (i.e. low FI vs high FI) and survey, a negative value means that the effect of fishing above the FI threshold (x-axis) was negative for a particular species. (e-f) Number of times the FI thresholds were selected in the models for epifaunal and infaunal abundance and presence/absence in each sediment type.

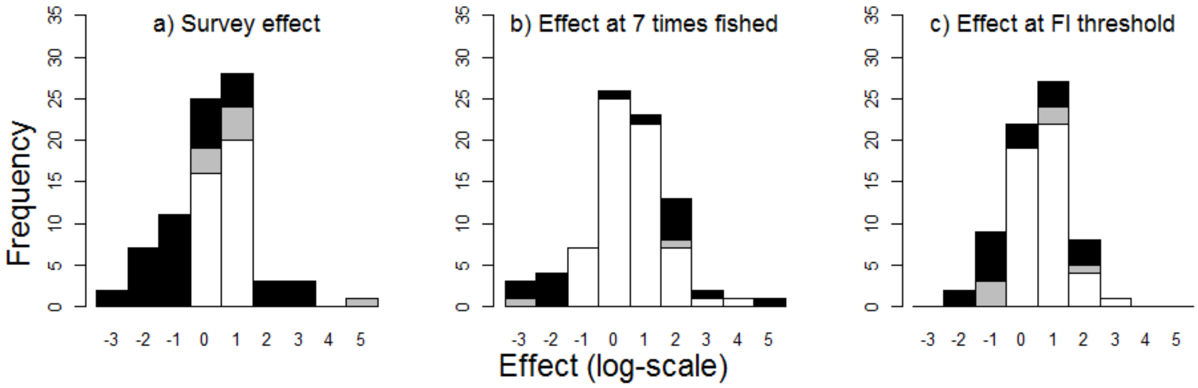

Figure S2.6. Histograms of the effect of seasonal changes at the taxon-level (a) compared to the fishing intensity effect after 4 months (b-c). The term effect here corresponds to the estimate reported from the models (i.e. log-scale percentage change from before to after fishing). With the continuous FI gradient, the level of fishing intensity was chosen to visually match the survey effect, i.e. 7 times fished (b). This was compared to the effect sizes identified with the threshold analysis (c). Each panel includes effects on abundance, biomass and presence of epifauna and presence of infauna. The colors indicate the level of significance: black is for p-value <0.05, grey for p-value ≥0.05 and <0.1, and no color for not-significant (≥0.1) (figure comparable to Leblanc et al. 2015).

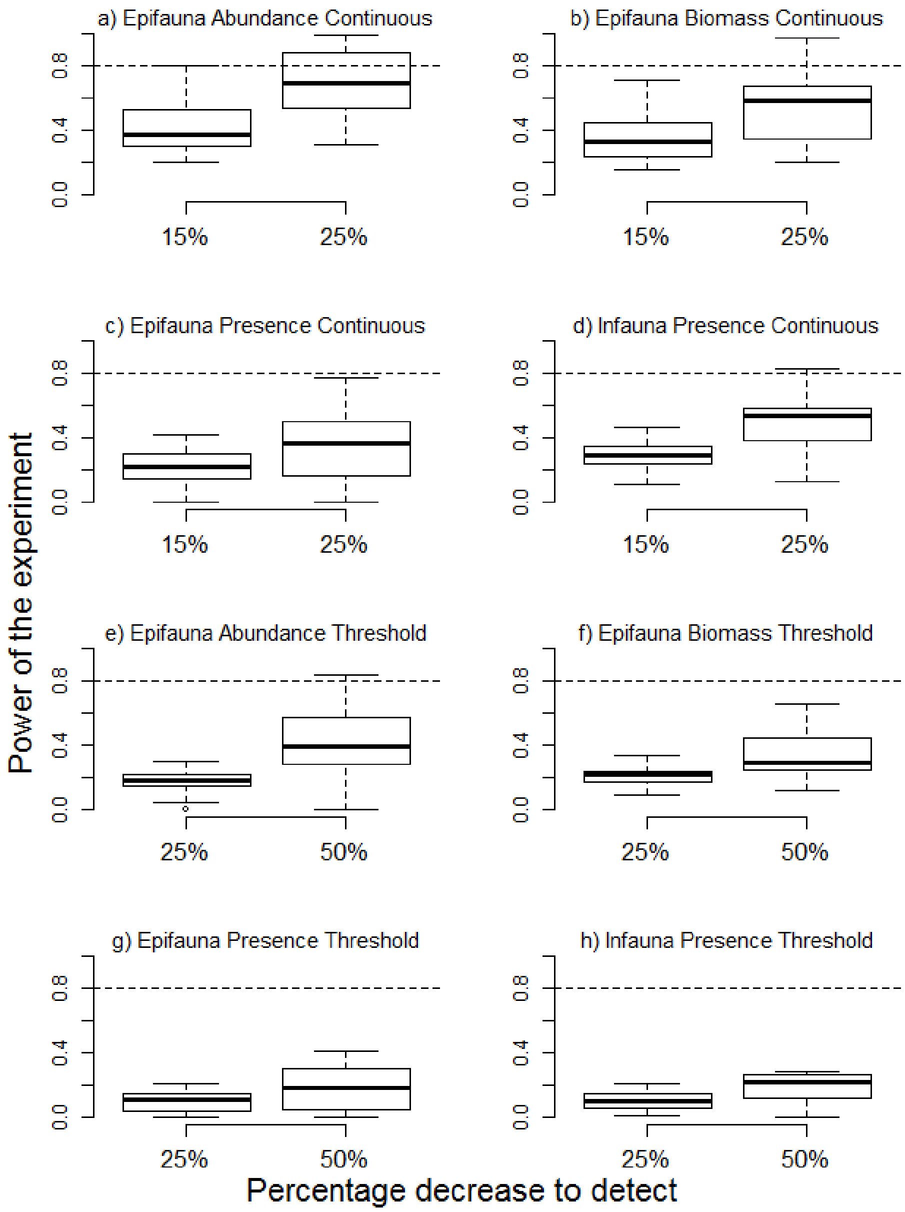

Figure S2.7. Detection power at the species (or family) level. The dash line represents 80% power to detect the change given on the x-axis. Results are given for the models with continuous fishing intensity (a-d) and the threshold models (e-h). Note that infauna abundance is not given as only one family was analyzed with abundance. Infauna biomass was only available at the group (class or phylum) level.
